# Replication and validation of two novel magnetoencephalography functional connectivity measures in Alzheimer’s disease

**DOI:** 10.1162/IMAG.a.1113

**Published:** 2026-01-27

**Authors:** Elliz P. Scheijbeler, Milo Molleson, Deborah N. Schoonhoven, Anne M. van Nifterick, Arjan Hillebrand, Willem de Haan, Cornelis J. Stam, Alida A. Gouw

**Affiliations:** Alzheimer Center Amsterdam, Department of Neurology, Amsterdam UMC location Vrije Universiteit Amsterdam, Amsterdam, The Netherlands; Amsterdam Neuroscience, Neurodegeneration, Amsterdam, The Netherlands; Department of Clinical Neurophysiology and MEG Center, Department of Neurology, Amsterdam UMC location Vrije Universiteit Amsterdam, Amsterdam, The Netherlands; Amsterdam Neuroscience, Brain Imaging, Amsterdam, The Netherlands; Amsterdam Neuroscience, Systems & Network Neuroscience, Amsterdam, The Netherlands

**Keywords:** magnetoencephalography, resting-state, functional connectivity, replication, validation, Alzheimer’s disease

## Abstract

Two novel magnetoencephalography (MEG) functional connectivity measures were recently shown to be superior to well-known measures of amplitude correlation and phase synchronization in detecting early stage neurophysiological abnormalities, particularly neuronal hyperexcitability, in a computational model of Alzheimer’s disease (AD). The reliability of these measures in empirical data remains to be evaluated. We investigated whether the Phase Lag Time (PLT) and Joint Permutation Entropy (JPE) could identify consistent patterns of between- and within-group functional connectivity in the theta (4–8 Hz), alpha (8–13 Hz), and beta (13–30 Hz) frequency bands in eyes-closed resting-state MEG recordings from two independent cohorts of AD patients (*n* = 28/*n* = 29) and control subjects (*n* = 29/*n* = 27). We assessed the classification performance of the measures and examined their construct validity using a computational approach. Results were compared with those obtained using two previously validated functional connectivity measures, the leakage-corrected Amplitude Envelope Correlation (AEC-c) and Phase Lag Index (PLI). In both cohorts, whole-brain analysis identified significant group differences (AD patients versus control subjects) in functional connectivity estimated by PLT and JPE across the theta and beta bands (effect sizes: 0.15–0.40; *p* < .05). Regional analysis revealed that 43–77 regions (out of 80) showed significant group differences in these frequency bands, with significant cross-cohort correlations between regional difference scores of *r_p_* = 0.37–0.55, *p* < .001. PLT and JPE connectivity matrices revealed strong within-group consistency across cohorts for both AD patients and control subjects in all frequency bands (*r_s_* > 0.80, *p* < .001). Logistic regression models trained on whole-brain PLT or JPE values in the theta or beta band achieved area under the curve values between 0.72 and 0.78. Finally, the PLT and JPE were shown to be sensitive to changes in coupling strength in a whole-brain computational model. Across all analyses, the PLT and JPE performed as well as, or better than, the AEC-c and PLI. The PLT and JPE can reliably detect neurophysiological abnormalities related to AD from eyes-closed resting-state MEG recordings. Since accumulating evidence identifies neuronal hyperexcitability as a key pathological process in early AD and a potential therapeutic target, these novel functional connectivity measures could play a valuable role in early disease detection and the evaluation of treatments aimed at restoring the excitation–inhibition balance in AD.

## 1 Introduction

Since the first recording of magnetic fields originating from the human brain in the 1960s (D. Cohen, 1968), increasingly complex analytic techniques have been applied to understand the spatial, temporal, and spectral structure of magnetoencephalography (MEG) signals. Rigorous testing, validation, and comparison of new methods to existing techniques are essential to ensure the credibility of research findings. While progress has been made in this regard, the reliability of many techniques for analyzing electrophysiological signals remains suboptimal (Cliff et al., 2023; Colclough et al., 2016; Liuzzi et al., 2017; Nagy et al., 2024).

A prominent feature of MEG signals is the presence of oscillatory activity across various frequency ranges, typically split into the delta (0.5–4 Hz), theta (4–8 Hz), alpha (8–13 Hz), beta (13–30 Hz), and gamma (30–48 Hz) bands (Baillet, 2017; Gross, 2019; F. Lopes da Silva, 2013). The synchronization of oscillatory activity is considered one of the important mechanisms for communication between brain regions (Brookes et al., 2011; Donner & Siegel, 2011; Fries, 2005; Hillebrand et al., 2012; Schnitzler & Gross, 2005; Tewarie et al., 2019). Inter-regional communication is, therefore, often investigated in terms of functional connectivity, which refers to the existence of statistical dependencies between the activity profiles of distinct brain regions (Aertsen & Preissl, 1991). Aberrant resting-state functional connectivity has been associated with several neurological disorders, including Alzheimer’s disease (AD) (Boon et al., 2017; Engels et al., 2017; Garcés et al., 2013; López-Sanz et al., 2017; Scheijbeler et al., 2022; Tewarie et al., 2014; van Dellen et al., 2014).

Accumulating evidence suggests that AD pathology initiates a cascade of biochemical and molecular changes that tip the excitation–inhibition (E-I) balance in the brain toward excessive excitation, thereby disturbing both local neuronal circuits and large-scale functional networks (Busche & Konnerth, 2016; Giorgio et al., 2024; Maestú et al., 2021; K. G. Ranasinghe et al., 2022a). Resulting alterations in brain network connectivity have been associated with cognitive dysfunction in AD (de Haan et al., 2012; Liu et al., 2014; Maestú et al., 2021; Pusil et al., 2019; Sui et al., 2015; J. Wang et al., 2015; X. Wang et al., 2021; Zhang et al., 2021) and have been linked to markers of AD pathology, both on PET imaging (Scheijbeler et al., 2025; Schoonhoven et al., 2023) and in plasma (Carrasco-Gómez et al., 2025; García-Colomo et al., 2024, 2025). One intriguing possibility is that, unlike structural damage, changes in functional connectivity may be reversible. It has been hypothesized that by restoring the E-I balance in the brain, for example, through pharmacological approaches (Isla et al., 2023; Vossel et al., 2021) or non-invasive brain stimulation (Menardi et al., 2022), neural circuits can regain the flexibility to effectively synchronize their activity patterns, thereby promoting normalized brain synchrony and potentially influencing cognitive outcomes.

Since it is impossible to directly assess the E-I ratio in the human brain using non-invasive approaches, MEG functional connectivity measures have been proposed as surrogate markers of E-I (Cuesta et al., 2022; K. G. Ranasinghe et al., 2022a; Stam & de Haan, 2024; Stam et al., 2023; van Nifterick et al., 2022). Compared with electroencephalography (EEG), MEG offers higher spatial resolution, enabling more accurate source reconstruction and more reliable estimation of functional connectivity (F. Lopes da Silva, 2013). This positions MEG as the preferred non-invasive method for investigating E-I balance. Two novel functional connectivity measures—the Phase Lag Time (PLT; Stam & de Haan, 2024) and the Joint Permutation Entropy (JPE; Scheijbeler et al., 2022)—were recently shown to be more sensitive to neuronal hyperexcitability than well-known measures of amplitude correlation (i.e., the leakage-corrected amplitude envelope correlation [AEC-c]; Brookes et al., 2012; Hipp et al., 2012) and phase synchronization (i.e., the Phase Lag Index [PLI]; Stam et al., 2007) in a whole-brain computational model of AD (Stam & de Haan, 2024). Another study showed that Aβ deposition was associated with a reduction of functional connectivity (estimated by JPE) over time in cognitively unimpaired adults (Scheijbeler et al., 2025), supporting the hypothesis that this functional connectivity measure is a sensitive marker of neuronal hyperexcitability. These findings suggest that the PLT and JPE could play a valuable role in early disease detection and the evaluation of treatments aimed at restoring E-I balance in AD. However, further evaluation of the reliability of these novel functional connectivity measures, particularly in empirical data, is needed.

The present study aimed to determine whether the PLT and JPE can identify consistent patterns of between- and within-group functional connectivity in the theta (4–8 Hz), alpha (8–13 Hz), and beta (13–30 Hz) bands in eyes-closed resting-state MEG recordings from two independent cohorts of AD patients and control subjects. First, we assessed whether whole-brain and regional differences in functional connectivity could be replicated across cohorts. Next, we compared within-group functional connectivity patterns across the independent cohorts. After merging the cohorts, receiver operating characteristic (ROC) curve analysis was conducted to assess the classification performance of the functional connectivity measures at single-subject level. Finally, we investigated the construct validity of the measures using a whole-brain computational model. Since computational models allow for the manipulation of specific parameters, such as the functional coupling strength between “brain regions”, we can assess how this would affect the functional connectivity measures. In a similar way, previous studies have established that the JPE and PLT are sensitive to changes in the E-I ratio (Stam & de Haan, 2024; van Nifterick et al., 2022). Results were compared with those obtained using two previously validated functional connectivity measures: AEC-c and PLI. Functional connectivity research in the AD field still partially relies on measures sensitive to spatial leakage confounds (Briels et al., 2020; Mandal et al., 2018; P. Ranasinghe & Mapa, 2024). In this study, however, we specifically chose to investigate and compare measures that are insensitive to these confounds, to avoid the associated problems (Brookes et al., 2012; Colclough et al., 2015; Hipp et al., 2012; Maldjian et al., 2014; S. Palva & Palva, 2012). Based on earlier findings, we anticipated that the AEC-c would produce best results in the alpha and beta bands, while the PLI, PLT, and JPE were expected to be particularly effective in the theta band (Briels et al., 2020; Scheijbeler et al., 2022, 2025; Schoonhoven et al., 2022). The PLT and JPE were hypothesized to outperform the AEC-c and PLI in terms of sensitivity to group differences and classification performance, as well as their ability to replicate results across independent cohorts.

## 2 Materials and Methods

### 2.1 Subjects

All subjects visited the memory clinic of the Amsterdam Alzheimer Center (Amsterdam, the Netherlands) between May 2015 and March 2018 and provided written informed consent for the use of their data for research purposes. Data were obtained from the Amsterdam Dementia Cohort (van der Flier & Scheltens, 2018). The standardized diagnostic procedure of the Amsterdam Alzheimer Center included medical history taking, neurological and neuropsychological examination, laboratory testing, MRI, MEG or EEG, and, if possible, a lumbar puncture to collect cerebrospinal fluid (CSF) and/or an Aβ positron emission tomography (PET) scan (van der Flier & Scheltens, 2018). Diagnoses were established in a multidisciplinary consensus meeting according to international guidelines (Jessen et al., 2014; McKhann et al., 2011). This study included patients with a diagnosis of subjective cognitive decline (SCD) or probable AD. SCD subjects were included as control subjects; they exhibited normal performance on standardized cognitive tests, had no psychiatric or neurological conditions, and were not using psychoactive medications. Probable AD patients were in the dementia stage of the disease, characterized by impairment on multiple cognitive domains. The presence of AD pathology was verified using CSF and/or Aβ-PET. When both Aβ-PET and CSF data were available, Aβ-PET (categorized as Aβ positive or negative based on visual read) was decisive. The cutoff for positive CSF Aβ 1–42 was set at 813 pg/ml (drift-corrected) (Tijms et al., 2018). CSF p-tau values were used to establish tau positivity, with a cutoff of 52 pg/ml (Mulder et al., 2010). Probable AD patients were excluded if they were Aβ negative, whereas control subjects were excluded if they were Aβ positive. For control subjects lacking biomarker information, follow-up visits were examined to verify whether their SCD diagnosis remained consistent. Subjects were divided into two cohorts of approximately equal size. Within each cohort, differences in sex, age, and education level were minimized between groups. This dataset has previously been analyzed and reported on by Schoonhoven et al. (2022). Demographic and clinical characteristics of the included subjects are given in Table 2.

### 2.2 MEG recording

MEG data were acquired in a magnetically shielded room using a 306-channel whole-head Vectorview MEG system (Elekta Neuromag Oy, Helsinki, Finland). Subjects were in supine position. The acquisition protocol consisted of two 5-min eyes-closed recordings. During recording, subjects were instructed to open and close their eyes several times to assess the reactivity of the alpha rhythm. Only eyes-closed data from the first recording were used for analysis. Subjects were instructed to relax but stay awake. Recordings were sampled at 1,250 Hz, with an anti-aliasing filter of 410 Hz and a high-pass filter of 0.1 Hz. The head position relative to the MEG sensors was recorded continuously using the signals from five head position indicator coils. The head-localization coil positions and outline of the subjects’ scalp (~500 points) were digitized using a 3D digitizer (Fastrak, Polhemus, Colchester, VT, USA).

### 2.3 MEG pre-processing

Channels containing excessive artifacts (such as flat, very noisy, and squid-jump channels) were visually identified and discarded from the raw data before applying the temporal extension of the signal space separation (tSSS) filter (MaxFilter software version 2.2.15 by Elekta Neuromag Oy; Taulu & Simola, 2006). The denoised signal was then reconstructed for all sensors (Taulu et al., 2004, 2005). The digitized scalp surface of each subject was co-registered to the best-matching template MRI (very small, small, medium or large, custom built using 3D T1-weighted MRI images from the Amsterdam Dementia Cohort) using surface matching with an estimated accuracy of approximately 4 mm (Whalen et al., 2008). Previous research reported no (systematic) bias or inconsistency for MEG functional connectivity measures when using template versus native MRIs for co-registration (Douw et al., 2018). A single sphere, fitted to the outline of the scalp as obtained from the co-registered MRI, was used as a volume conductor model for the beamforming approach described below.

An atlas-based beamforming approach (Hillebrand et al., 2012) was used to obtain source-localized activity. For a detailed description, we refer the reader to Hillebrand et al. (2016). The broadband (0.5–100 Hz) sensor level time series were projected through the normalized beamformer weights to reconstruct time series of neuronal activity for 80 regions of interest (parcels), as included in the Automated Anatomical Labelling (AAL) atlas (Gong et al., 2009; Tzourio-Mazoyer et al., 2002; Supplementary Table S1). This included 78 cortical regions and both hippocampi. The source-reconstructed time series were converted to ASCII format and downsampled to 312 Hz to reduce computational load while preserving the relevant frequency content of the MEG signals. For each recording, 10 non-overlapping epochs containing 4,096 samples (13.1 s) of eyes-closed, artifact-free data were selected from the first eyes-closed recording, based on visual inspection by an experienced clinical neurophysiologist (A.G.). Epoch length was determined based on evidence that phase- and amplitude-based connectivity measures stabilize for epochs ≥12 s (Fraschini et al., 2016) and because FFT-based processing is most efficient when applied to signals with a length equal to a power of 2 (4,096 = 2¹²). All epochs received a quality score of 1 to 4: 1 = no eye movement, muscle artifacts, signs of drowsiness, or other artifacts; 2 = minimal presence of artifacts; 3 = moderate presence of artifacts; and 4 = strong presence of artifacts. The 10 epochs with the highest quality score were subsequently selected for each subject. For a more detailed description of the selection method, see Gouw et al. (2017).

### 2.4 Time series analysis

As a first step, the time series were digitally filtered into the theta (4–8 Hz), alpha (8–13 Hz), and beta (13–30 Hz) frequency bands using a digital band-pass FFT filter. Delta and gamma frequencies were excluded from analysis because of the low reliability of AEC-c and PLI findings in these frequency bands (Briels et al., 2020, Schoonhoven et al., 2022). Next, the analytic signal was derived using the Hilbert transform, as described previously (Stam et al., 2007, 2023). This complex signal provides information about the instantaneous amplitude and phase of the original time series. Pairwise functional connectivity (estimated using AEC-c, PLI, PLT, and JPE) was estimated between all 80 parcels defined in the AAL atlas, resulting in an 80 × 80 connectivity matrix for each subject and each measure. Next, for each parcel, its connectivity strength to the rest of the brain was obtained by averaging its 79 pairwise connectivity values. This average corresponds to the concept of “weighted degree” or “node strength” in graph theory. Finally, whole-brain functional connectivity was calculated as the mean connectivity strength across all 80 parcels.

To improve the stability of the connectivity estimates (both whole-brain and regional), functional connectivity values were computed separately for each epoch (10 per subject) and then averaged within each subject prior to group-level statistical analyses.

### 2.5 Functional connectivity measures

The technical definitions of the functional connectivity measures described below are given in Supplementary Table S2.

#### 2.5.1 Leakage-corrected amplitude envelope correlation

The amplitude envelope correlation (AEC) is defined as the Pearson correlation of the amplitude envelopes of pairs of time series (Brookes et al., 2011; Bruns et al., 2000; Hipp et al., 2012). Prior to AEC estimation, pair-wise orthogonalization was performed to correct for the effects of volume conduction or field spread (referred to as signal leakage in source space) (Brookes et al., 2012; Colclough et al., 2015). Orthogonalization separates signals into distinct, independent components. By only analyzing non-overlapping components, the unique contribution of each brain region is isolated and potential distortions introduced by volume conduction are discarded. Leakage-corrected AEC, or AEC-c, values were normalized between 0 and 1, with 0.5 indicating no functional connectivity (Briels et al., 2020; Schoonhoven et al., 2022).

#### 2.5.2 Phase lag index

The instantaneous phase of two time series indicates their temporal relationship to each other (Stam & van Straaten, 2012). The phase lag index (PLI) reflects the level of phase synchronization between pairs of time series (Stam et al., 2007). In the absence of phase synchronization between two time series, their phase difference distribution should be uniform. Deviations from this uniform distribution suggest the presence of phase synchronization. The PLI quantifies this asymmetry in the distribution. It ranges from 0 to 1, where 0 indicates no connectivity or only zero-lag phase locking and 1 signifies perfect non-zero-lag phase locking. The measure is not sensitive to volume conduction/field spread, as it excludes phase differences centered around 0 mod π.

#### 2.5.3 Phase lag time

**Fig. 1.**
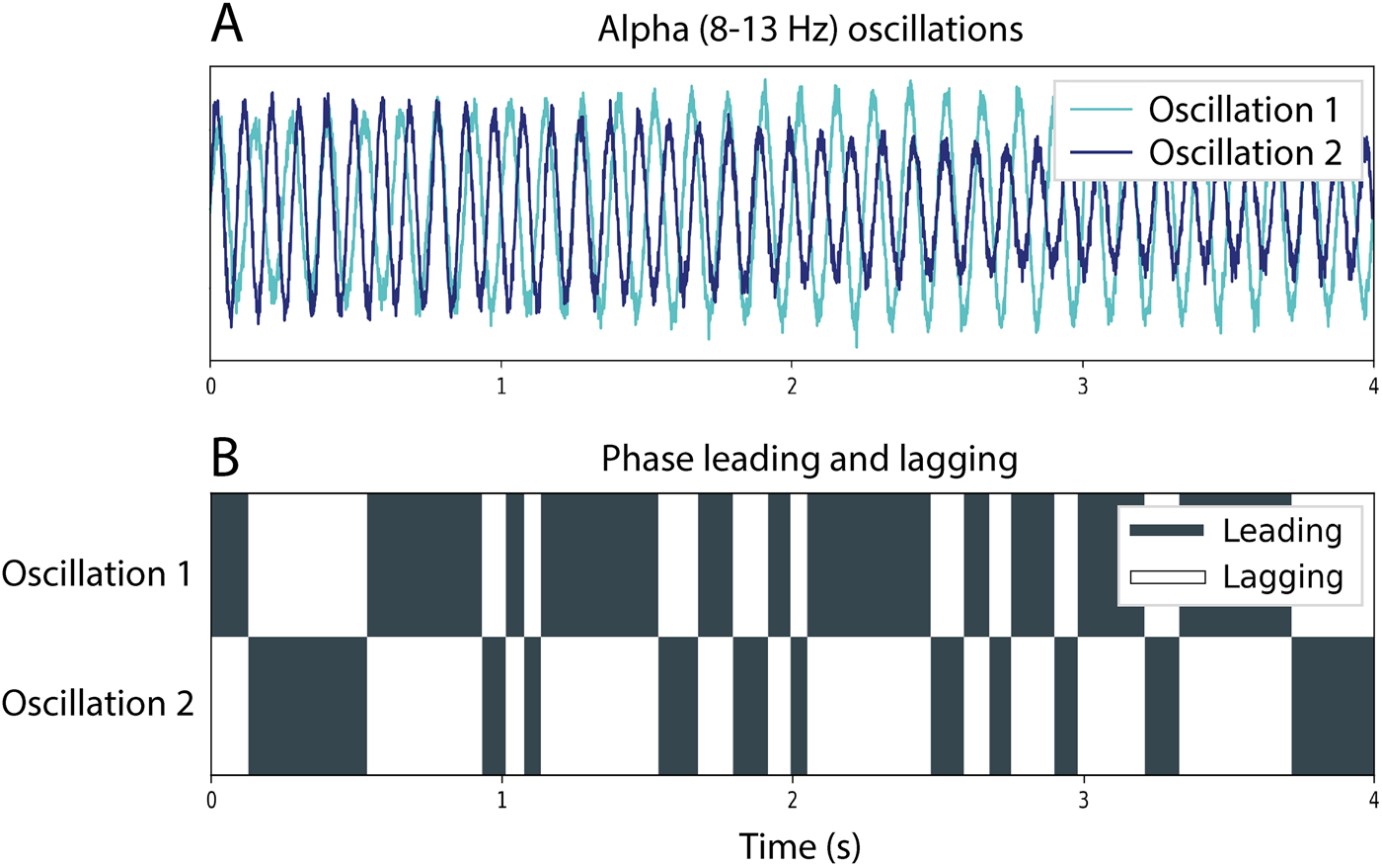
Diagram visualizing the dynamic, sub-second nature of phase leading and lagging. (A) Two superimposed oscillatory signals filtered in the alpha (8–13 Hz) band, with one oscillation in cyan and the other in blue. (B) Phase leading and lagging roles fluctuate rapidly between time series over time. The x-axis represents time in seconds, while the y-axis displays two rows, each corresponding to one of the oscillatory signals (1 or 2). Dark blocks indicate periods when one oscillation is leading, while light blocks denote periods when it is lagging. Reversals in leading/lagging roles occur on a sub-second timescale. The PLT estimates the average time between two successive phase reversals.

When comparing two oscillatory time series, one can be described as leading (ahead), while the other is lagging (falling behind). While the PLI is able to capture the static, non-zero phase difference between two time series, it does not capture the rapid fluctuations in leading/lagging roles (“fragile binding”) between time series that can occur on a sub-second timescale (Fig. 1). A reversal in leading/lagging roles is known as a phase reversal or zero-crossing, at which the phase difference Δφ(*t*) = 0 (Stam et al., 2007). The phase lag time (PLT) estimates the average time between two successive phase reversals (in seconds), controlling for the sample frequency of the time series (Stam & de Haan, 2024). A longer average time between phase reversals (i.e., more stable leading and lagging roles) indicates stronger phase synchronization, or functional connectivity. In the case of volume conduction/field spread, the phase difference and PLT will be zero. Important to note is that the PLT, in contrast to the PLI, does not depend upon epoch length (Fraschini et al., 2016).

#### 2.5.4 Joint permutation entropy

The Joint Permutation Entropy (JPE) is a functional connectivity measure that captures the variability of local signals, as well as their interactions. For a detailed description of the measure, see Scheijbeler et al. (2022) and Yin et al. (2019). Computation of the JPE begins by transforming the (filtered) time series from each parcel into a sequence of discrete ordinal patterns or symbols. Each pattern includes *n* amplitude values separated by a time lag τ. Similar to previous studies, we used a time lag of 1 and a pattern length of 4 (Scheijbeler et al., 2022, 2025; Stam & de Haan, 2024). The *n* amplitudes in each pattern are then ranked from highest (1) to lowest (*n*), resulting in *n*! different ordinal patterns. For two different signals, there are *n*! * *n*! possible combinations of patterns. To correct for the influence of volume conduction/field spread, symmetric and anti-symmetric patterns in the two time series are excluded (King et al., 2013). The JPE is computed as the Shannon information entropy of the resulting matrix of co-occurring ordinal patterns. If the co-occurrence of ordinal patterns in the two time series is highly variable (i.e., weak functional connectivity), the JPE value will be high. Conversely, if the co-occurrence of ordinal patterns in the two time series is regular and predictable (i.e., strong functional connectivity), the JPE value will be low. The JPE was normalized between 0 and 1 by dividing it by its maximum value. To facilitate comparison with more conventional connectivity measures, we report inverted JPE values throughout the paper, so that higher values correspond to stronger (less variable) functional connectivity.

### 2.6 Computational modeling of whole-brain dynamics

To investigate whether the functional connectivity measures under investigation are capable of detecting true interactions between brain regions, which they are designed to do, we employed a computational modeling approach. The brain was modeled as a network of 78 cortical regions, as included in the AAL atlas. For each region, MEG-like oscillatory activity was simulated using a neural mass model. The 78 neural mass models were coupled based on an average human structural connectivity matrix derived from diffusion tensor imaging (Gong et al., 2009).

Neural mass models are based on the mean-field assumption, which posits that the collective behavior of a homogeneous population of neurons can be described by a single time-dependent variable. While neural mass models were originally introduced to explain the alpha rhythm (F. H. Lopes da Silva et al., 1974; Wilson & Cowan, 1972; Zetterberg et al., 1978), they have since been used to investigate various normal and pathological EEG and MEG phenomena (David & Friston, 2003; de Haan et al., 2012; Ponten et al., 2013; K. G. Ranasinghe et al., 2022b; Stam et al., 1999; van Nifterick et al., 2022). A single neural mass model consists of coupled populations of excitatory and inhibitory neurons. The excitatory and inhibitory populations are characterized by their average membrane potentials and firing rates (spikes/s). Incoming spikes are translated into changes in the average membrane potential through an impulse response function. The average membrane potential is subsequently converted to an outgoing firing rate with a sigmoidal function.

In this study, we used the following impulse response function:

h(τ)=A[exp(−aτ)−exp(−bτ)]   for τ≥0

h(τ)=0                                      for τ<0,

with associated sigmoid functions S1(x) and S2(x), defined as:

S[Vm−Vd]=gexp{q(Vm−Vd)}             for Vm≤Vd

S[Vm−Vd]=g[2−exp{q(Vd−Vm)}]    for Vm>Vd.

**Fig. 2.**
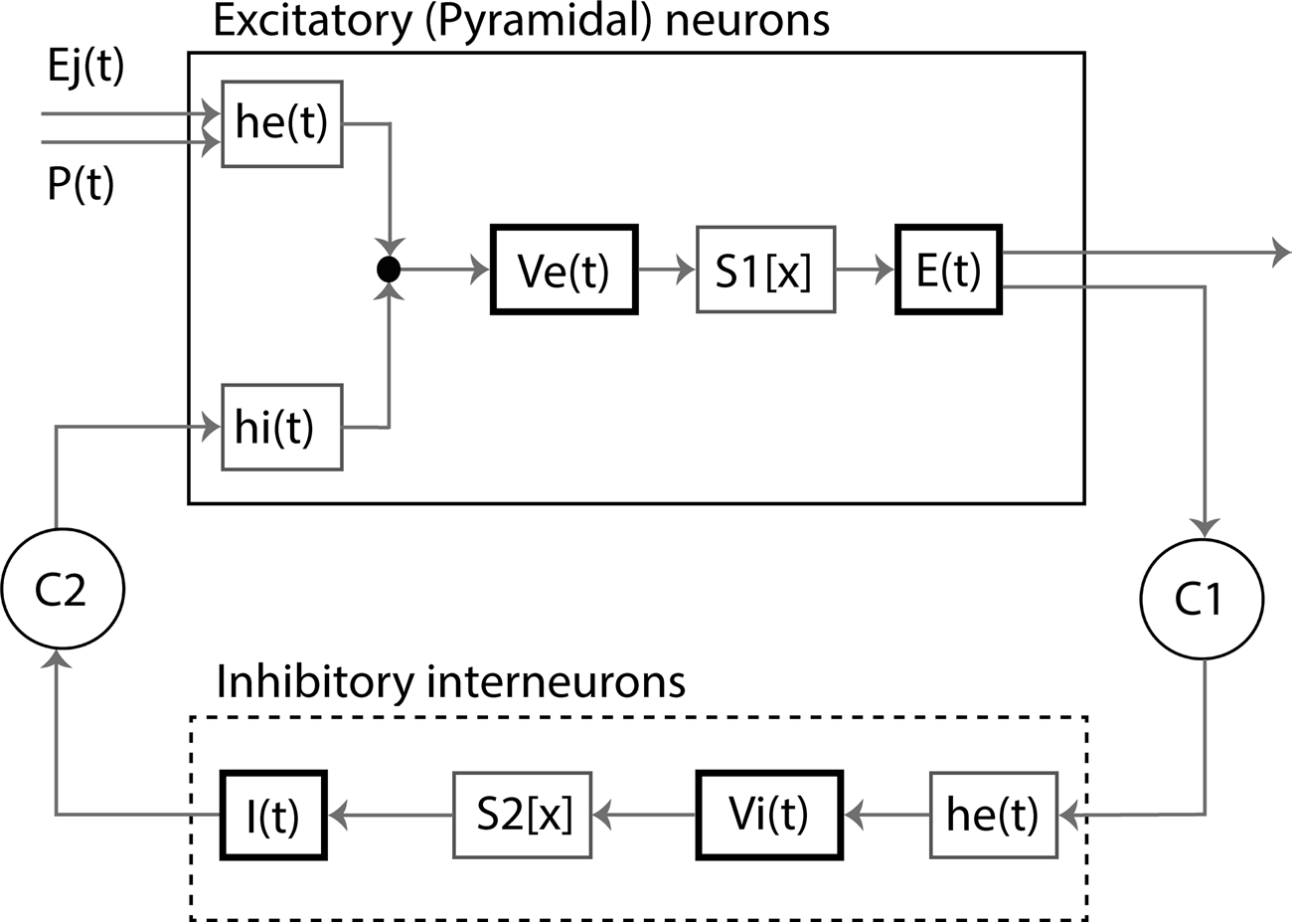
Schematic representation of a single neural mass model. The upper rectangle represents a population of excitatory neurons, the lower rectangle a population of inhibitory interneurons. Each population is characterized by an average membrane potential [*Ve*(t) and *Vi*(t)] and firing rate [*E*(t) and *I*(t)]. Membrane potentials are converted to firing rates by sigmoid functions *S1*[x] and *S2*[x]. Firing rates are converted to membrane potentials by impulse response functions he(t) and hi(t). *C1* and *C2* are coupling strengths between the two populations. *P*(t) and *Ej*(t) are excitatory firing rates coming from the thalamus or other excitatory neuronal populations.

All functions were taken from Zetterberg et al. (1978). The excitatory population of each neural mass model receives external input from the thalamus and other populations. The time series of the average membrane potential of the excitatory population serves as the model’s output and is assumed to reflect MEG-like activity. A schematic representation of a single neural mass model is shown in Figure 2. Further mathematical details of the neural mass model can be found in previous papers (de Haan et al., 2012; Ponten et al., 2010; van Dellen et al., 2013).

Neural mass models can be coupled by using the outgoing firing rate of one model as input to another (*E_j_ (t)* in Fig. 2), with the interaction mediated by a coupling strength, *S*. We systematically varied the global coupling strength, *S*, between neural mass models from 0 to 2.0 in increments of 0.5, progressively increasing the excitatory interaction between “regions” in the network. We then assessed how these changes in network dynamics affected whole-brain functional connectivity.

For each value of coupling strength *S*, 20 epochs of 4,096 samples were generated at a sampling frequency of 500 Hz. The 5,000 samples preceding the first epoch were discarded to ensure the system had reached a stable state. Whole-brain functional connectivity (estimated by AEC, PLI, PLT, and JPE) was calculated as a mean over the 78 cortical regions for the individual epochs, after which results were averaged over epochs. For each value of coupling strength *S* greater than 0, we report the percentage change (mean ± standard deviation) in AEC-c, PLI, PLT, and JPE relative to the functional connectivity values at *S* = 0, to ensure a consistent scale across measures. The simulated MEG data were not filtered into specific frequency bands prior to computing functional connectivity, as the analysis focused on the relationship between coupling and functional connectivity, rather than on frequency-specific effects. In addition, the selected model parameter settings (given in Table 1) caused the Lopes da Silva model to predominantly generate alpha oscillations, hence filtering in the theta or beta band may produce atypical results (F. H. Lopes da Silva et al., 1974). Supplementary Figure S1 shows the simulated MEG signals and their corresponding whole-brain average power spectra for different levels of global coupling strength *S*. Note that we computed the uncorrected AEC for the simulated MEG data, as volume conduction/field spread is not a concern in this scenario.

**Table 1.** Overview of model parameters: Descriptions and (range of) values.

Parameter	Interpretation	Value
Sample frequency		500 Hz
Sample time		2 ms
Epoch length		4,096 samples
Pt	Thalamic input	550 spikes/s
Noise level	Fluctuations of thalamic input around mean	1.0
Amp1	Amplitude of EPSP	1.6 mV
Amp2	Amplitude of IPSP	32 mV
A1	Shape parameter of EPSP	55 s-1
B1	Shape parameter of EPSP	605 s-1
A2	Shape parameter of IPSP	27.5 s-1
B2	Shape parameter of IPSP	55 s-1
G	Shape parameter of sigmoidal function relating membrane potential to firing rates	25 s-1
Q	Shape parameter of sigmoidal function relating membrane potential to firing rates	0.34 mV-1
Vd1	Threshold potential for converting membrane potential to firing rates for excitatory neurons	7 mV
Vd2	Same as above for inhibitory neurons	7 mV
C1	Connection strength excitatory to inhibitory populations	32
C2	Connection strength inhibitory to excitatory populations	3
S	Coupling strength between neural masses	0/0.5/1/1.5/2

### 2.7 Statistical analysis

Differences in demographic and clinical variables between AD patients and control subjects, as well as between cohorts, were evaluated using independent samples t-tests, Mann–Whitney U tests, or chi-square tests, as appropriate. Statistical significance was set at *p* < .05.

#### 2.7.1 Whole-brain and regional group differences in functional connectivity

Non-parametric permutation tests (10,000 iterations) were used to compare functional connectivity (estimated by AEC-c, PLI, PLT, and JPE) between AD patients and control subjects across the whole brain, and for each of the 80 parcels individually. Group differences were evaluated independently in both cohorts. To evaluate the replication across cohorts, we compared several metrics: the effect size and significance of the whole-brain analysis in both cohorts (estimated by Cohen’s *d*; J. Cohen, 2013), the number of overlapping regions that showed significant group differences in both cohorts, and the magnitude and significance of the Pearson correlation of regional difference scores between the two cohorts. Regional difference scores were calculated by subtracting the average connectivity value of AD patients in cohort 1 from the average connectivity value of control subjects in cohort 1 for each of the 80 parcels. This process was then repeated for cohort 2, resulting in a separate set of 80 regional difference scores. The Pearson correlation was subsequently computed between the regional difference scores from cohort 1 and those from cohort 2. This correlation provides insight into the consistency of the spatial pattern of group differences across the two cohorts, even for regions that do not meet the significance threshold. In contrast, the overlap in significant regions indicates whether specific areas consistently exhibit group differences, suggesting a robust biological or clinical basis rather than results driven by cohort-specific variations or noise.

#### 2.7.2 Within-group functional connectivity

To evaluate the replication of within-group functional connectivity patterns across cohorts, we computed the Spearman correlation between the upper triangle of the (symmetric) average functional connectivity matrices from cohort 1 and the upper triangle of the average matrices from cohort 2.

#### 2.7.3 Multiple comparison correction

Multiple comparison correction was applied using false discovery rate (FDR) correction where appropriate (Benjamini & Hochberg, 1995). Specifically, within-group functional connectivity matrix correlations were FDR corrected, as were correlations performed between the regional functional connectivity difference scores (controls – AD) across cohorts. Analyses conducted independently within each cohort were not subjected to multiple comparison correction, as independent replication across datasets served as a safeguard against false-positive findings. By validating results in the second cohort, we focused on confirming the reliability of the findings through independent replication rather than relying on adjustments within a single dataset.

#### 2.7.4 ROC curve analysis

To determine the classification performance of the different functional connectivity measures at single-subject level, we used simple logistic regression models with group (AD patient versus control subject) as dependent variable and whole-brain AEC-c, PLI, PLT, or JPE values in the theta, alpha, or beta band as independent variables. ROC curves were plotted, and the area under the ROC curve (AUC) was reported with 95% confidence intervals. For this analysis, the two cohorts were merged into a single cohort to enhance statistical power.

### 2.8 Software

Time series analyses, including the discrete FFT, Hilbert transform, and functional connectivity estimation, as well as simulation of MEG data, were conducted using BrainWave software (version 0.9.136.26, available from https://github.com/CornelisStam/BrainWave). Statistical analysis was performed using BrainWave software, R studio version 4.2.1 and Matlab R2018b version 9.5.0.944444.

## 3 Results

### 3.1 Demographic and clinical characteristics

A total of 113 subjects were included in this study. Subjects were assigned to cohort 1 (*n* = 57) or cohort 2 (*n* = 56) (Table 2). Cohort 1 included 28 AD patients and 29 control subjects, while cohort 2 consisted of 29 AD patients and 27 control subjects. In both cohorts, control subjects were significantly younger than the AD patients. The groups (by design) also differed significantly in terms of Mini Mental State Examination (MMSE) score (Folstein et al., 1975), Aβ positivity, and tau positivity. Biomarker confirmation was available for all AD patients. For 10 control subjects (3 in cohort 1 and 7 in cohort 2), biomarker status was not available, due to either refusal or inability to undergo a lumbar puncture and/or an Aβ PET scan. Of these, six subjects underwent at least one clinical follow-up including neuropsychological testing after a minimum follow-up period of 2 years, which confirmed the absence of cognitive impairment.

**Table 2.** Demographic and clinical characteristics.

	Cohort 1 (*n* = 57)		Cohort 2 (*n* = 56)	
	AD (*n* = 28)	Controls (*n* = 29)	AD (*n* = 29)	Controls (*n* = 27)
Age (years)	66.3 ± 7.4*	56.7 ± 8.4	64.0 ± 6.8*	56.3 ± 10.6
Sex female, (*n*, (%))	16 (57)	11 (38)	16 (55)	11 (41)
Symptom duration (years)^a^	3.3 ± 2.3	3.5 ± 2.6	2.7 ± 1.6	3.3 ± 2.4
Education level (median, (IQR))^b^	5 (3–7)	6 (3–7)	5 (3–7)	5 (3–7)
MMSE score (median, (IQR))	20 (12–28)*	28 (26–30)	22 (15–29)*	27 (24–30)
Cerebrospinal fluid	*n* = 23	*n* = 19	*n* = 26	*n* = 18
Aβ 1–42 positive (*n*, (%))	23 (100)*	0 (0)	26 (100)*	0 (0)
p-tau positive (*n*, (%))	19 (83)*	6 (32)	23 (88)*	5 (28)
Aβ-PET	*n* = 14	*n* = 16	*n* = 7	*n* = 5
Positive (*n*, (%))	14 (100)*	0 (0)	7 (100)*	0 (0)

Shown are mean ± SD unless specified otherwise. Cutoffs for positivity were set at 813 pg/ml for CSF Aβ 1–42 (Tijms et al., 2018) and 52 pg/ml for CSF p-tau (Mulder et al., 2010). Aβ-PET scans were categorized as positive or negative based on visual read (Buckley et al., 2017).

a Time between the start of symptoms and diagnosis.

b Education level according to Verhage score (range 1–7).

* *p* < .05.

### 3.2 Whole-brain differences in functional connectivity between AD patients and control subjects

**Fig. 3.**
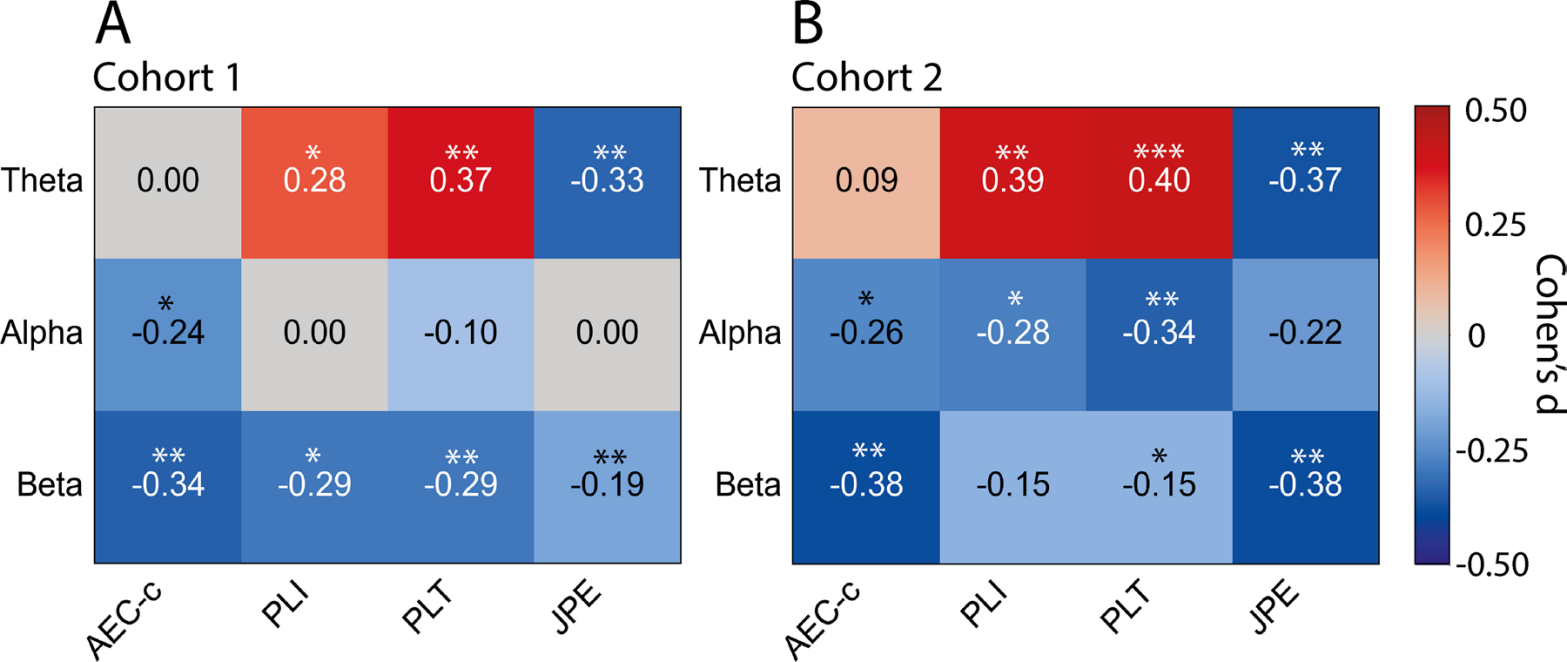
Whole-brain differences in functional connectivity between AD patients and control subjects in two independent cohorts. Whole-brain group differences are displayed as effect sizes (Cohen’s *d*) for each functional connectivity measure (AEC-c, PLI, PLT, and JPE) in each frequency band (theta (4–8 Hz), alpha (8–13 Hz), and beta (13–30 Hz)). (A) Results for cohort 1. (B) Results for cohort 2. Warmer colors (reds) indicate higher levels of functional connectivity in AD patients compared with control subjects, while cooler colors (blues) indicate lower levels of functional connectivity. *p < .05; **p < .01;***p < .001.

Figure 3 shows the effect sizes (Cohen’s *d*) and significance levels of the differences in whole-brain functional connectivity observed between AD patients and control subjects in cohort 1 and cohort 2. A detailed description of the results, including group means, standard deviations, and *p*-values, is given in Supplementary Table S3.

All functional connectivity measures revealed significant and replicable whole-brain group differences, although not in each frequency band. Whole-brain AEC-c values were significantly lower in AD patients than in control subjects in the alpha (*d* = -0.24, *d* = -0.26; for cohort 1 and cohort 2, respectively) and beta bands (*d* = -0.34, *d* = -0.38). Whole-brain PLI (*d* = 0.28, *d* = 0.39) and PLT values (*d* = 0.37, *d* = 0.40) were consistently higher in the theta band in AD patients, while JPE values were lower in this same frequency band (*d* = -0.33, *d* = -0.37). Finally, both PLT (*d* = -0.29; *d* = -0.15) and JPE values (*d* = -0.19; *d* = -0.38) were significantly lower in the beta band in AD patients compared with control subjects. Significantly lower PLI values in the beta band (*d* = -0.29) (observed in AD patients in cohort 1), as well as lower PLI (*d* = -0.28) and PLT values (*d* = -0.34) in the alpha band (observed in AD patients in cohort 2), could not be replicated. Across cohorts, the largest average effect sizes were observed for PLT theta (*d* = 0.39), AEC-c beta (*d* = -0.36), and JPE theta (*d* = -0.35), all of which were of moderate magnitude.

### 3.3 Regional differences in functional connectivity between AD patients and control subjects

**Fig. 4.**
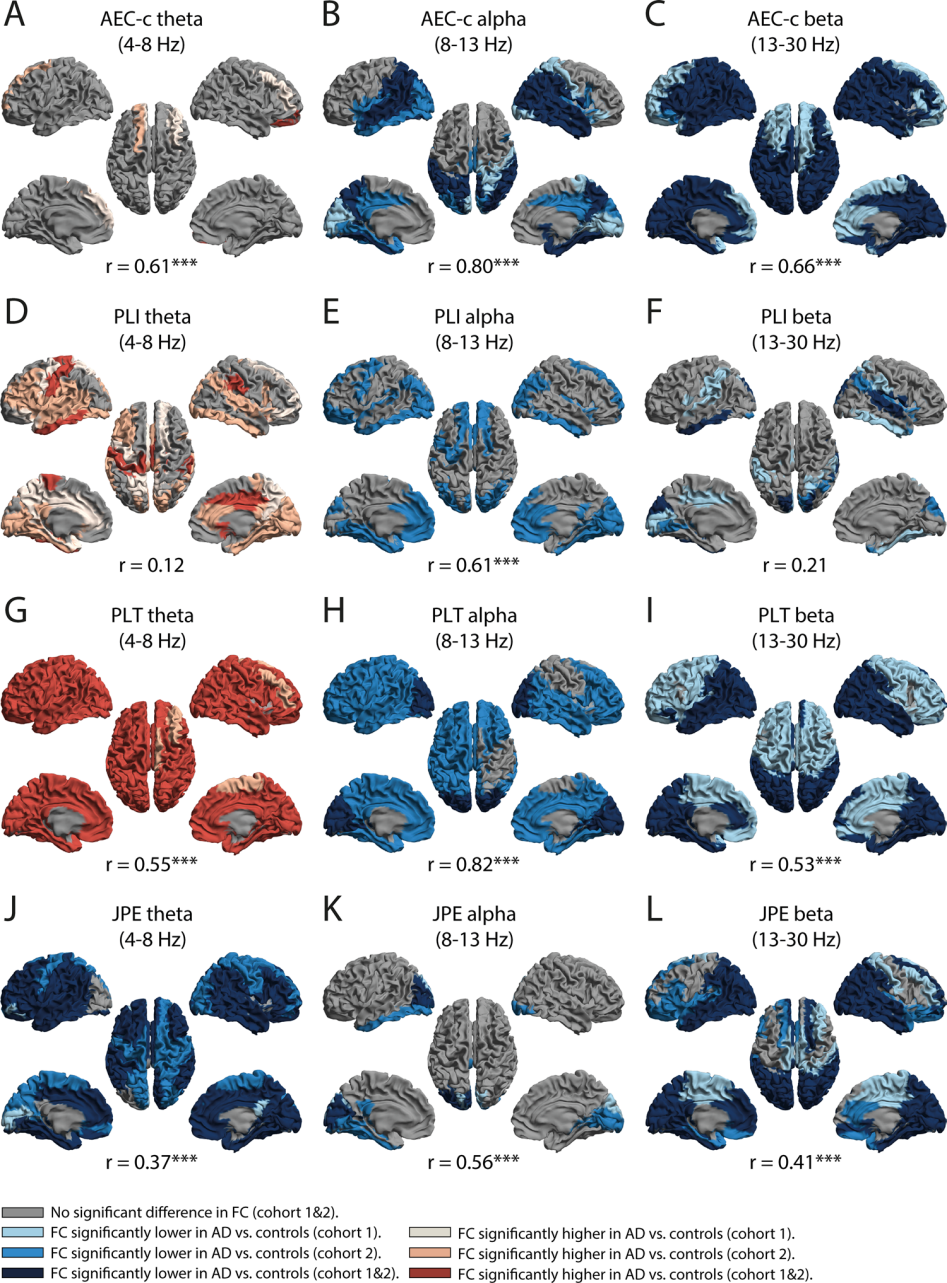
Regional differences in functional connectivity between AD patients and control subjects in two independent cohorts. Brain plots illustrate regional differences in functional connectivity (FC) between Alzheimer’s disease (AD) patients and control subjects across two independent cohorts. Regions showing higher FC in AD patients than in control subjects are highlighted in red, while regions with lower FC are shown in blue. Different shades indicate whether the effect was observed in cohort 1, cohort 2, or replicated in both cohorts. Each panel also includes the Pearson correlation (r) between the regional difference scores from both cohorts, reflecting the spatial consistency of group differences across cohorts. Regional differences are shown at *p* < .05. Differences in FC based on AEC-c (A–C), PLI (D–F), PLT (G–I), and JPE (J–L) in the theta, alpha, and beta bands. ***p < .001 (FDR corrected).

Figure 4 shows which cortical regions showed significant differences in functional connectivity between AD patients and control subjects in cohort 1, cohort 2, or both cohorts. The figure also shows the Pearson correlation between the regional difference scores (control subjects – AD patients) from cohort 1 and cohort 2.

We report few significant regional differences in AEC-c theta between AD patients and control subjects in both cohorts. The only replicable finding across cohorts was that AD patients demonstrated a significantly higher AEC-c value in the theta band for the right superior frontal orbital cortex (Fig. 4A). A larger number of regions, primarily in parieto-occipital and temporal areas, showed significantly lower AEC-c values in the alpha band in AD patients than control subjects. This finding was replicated across cohorts for 27 regions (Fig. 4B). Regional differences in AEC-c were best replicated in the beta band, with 59 cortical regions and both hippocampi showing significantly lower AEC-c beta values in AD patients than in control subjects (Fig. 4C).

Regional differences in functional connectivity estimated by PLI did not replicate well across cohorts. While AD patients exhibited higher PLI theta, lower PLI alpha, and lower PLI beta values compared with control subjects in several regions (Fig. 4D–F), these results could only be replicated for seven regions in the theta band and eight regions in the beta band. Regional differences in PLI alpha could not be replicated.

In contrast, significant group differences in functional connectivity estimated by PLT and JPE in the theta and beta bands were replicated across cohorts for more than half of the parcels (Fig. 4G, I, J, L). The PLT and JPE yielded most consistent results in the theta band. In both cohorts, 77 parcels exhibited significantly higher PLT theta values, and 55 parcels showed significantly lower JPE theta values in AD patients compared with control subjects. Replication of group differences proved more difficult in the alpha band, with only a few occipital parcels showing consistently lower PLT (*n* = 12) and JPE (*n* = 3) values in AD patients compared with control subjects (Fig. 4H, K). Consistent with the whole-brain results, PLT theta, AEC-c beta, and JPE theta demonstrated the largest and best replicable group differences in regional functional connectivity.

We found significant correlations between the regional difference scores from cohort 1 and cohort 2 for the AEC-c, JPE, and PLT across all frequency bands (*p* < .001; Fig. 4). The PLI only revealed a significant correlation between the regional difference scores from cohorts 1 and 2 in the alpha band. To ensure that the reported correlations reflected the similarity in the spatial distribution of group differences across cohorts, rather than overall group similarity, we shuffled the difference scores and reassessed the correlations. All correlations dropped significantly (*p* > .05), confirming that they were driven by spatial structure. For all four functional connectivity measures, the strongest correlations were demonstrated in the alpha band, with the highest correlations observed for PLT alpha (*r* = 0.82) and AEC-c alpha (*r* = 0.80).

### 3.4 Within-group functional connectivity

**Fig. 5.**
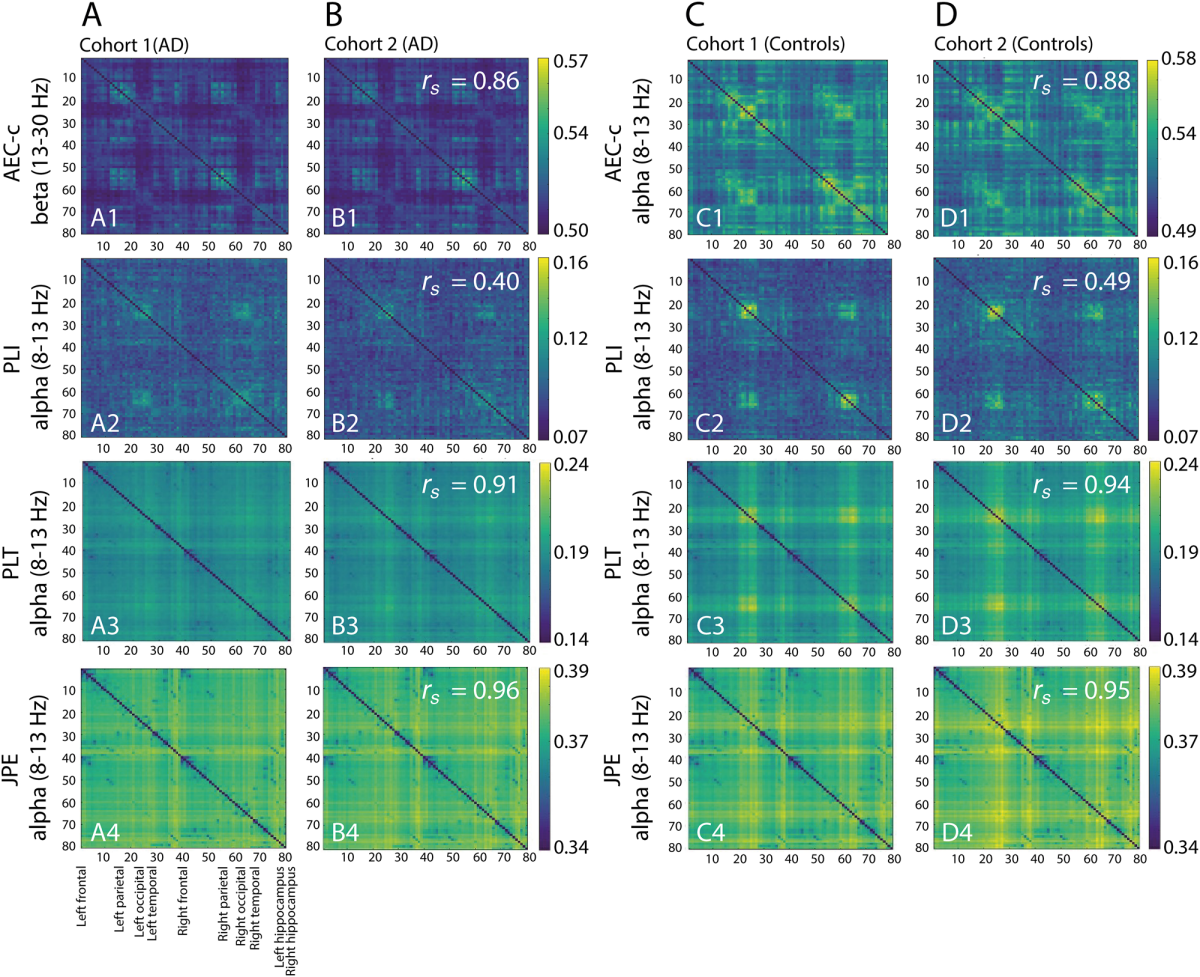
Average functional connectivity matrices of AD patients and control subjects in two independent cohorts. (A1–A4) Average AEC-c, PLI, PLT, and JPE matrices of AD patients in cohort 1. (B1–B4) Average AEC-c, PLI, PLT, and JPE matrices of AD patients in cohort 2. The Spearman correlation coefficients in the top right corner of each matrix quantify the consistency of connectivity patterns between both cohorts for the AD patient group. (C1–C4) Average AEC-c, PLI, PLT, and JPE matrices of control subjects in cohort 1. (D1–D4). Average AEC-c, PLI, PLT, and JPE matrices of control subjects in cohort 2. The Spearman correlation coefficients in the top right corner of each matrix quantify the consistency of connectivity patterns between both cohorts for the control group. For each functional connectivity measure, only the frequency band that exhibited the highest Spearman correlation between cohorts is presented. Supplementary Figures S2–S5 show a comprehensive overview of the matrices in all frequency bands. Regions of interest (1–80) are ordered from the left to the right hemisphere (see Supplementary Table S1). All Spearman correlations had a significance level of *p* < .001 (FDR corrected).

Replication of within-group functional connectivity patterns across cohorts was evaluated by computing the Spearman correlation between the average functional connectivity matrices from cohort 1 and the average functional connectivity matrices from cohort 2. Figure 5 shows, for each functional connectivity measure, the matrices from the frequency band that exhibited the highest Spearman correlation within each group (AD patients and control subjects). Supplementary Figures S2–S5 show a comprehensive overview, displaying the average functional connectivity matrices for AD patients and control subjects in both cohorts across all measures and frequency bands.

For the PLI, PLT, and JPE, highest correlations were consistently found in the alpha band, both for AD patients (PLI *r_s_* = 0.40; PLT *r_s_* = 0.91; JPE *r_s_* = 0.96) and control subjects (PLI *r_s_* = 0.49; PLT *r_s_* = 0.94; JPE *r_s_* = 0.95). For the AEC-c, the highest correlation was observed in the alpha band for control subjects (*r_s_* = 0.88), but in the beta band for AD patients (*r_s_* = 0.86). All correlations had a significance level of *p* < .001.

The PLT and JPE matrices demonstrated strong within-group consistency for both AD patients and control subjects in all frequency bands (*r_s_* > 0.80; range: 0.81–0.96; Supplementary Figs. S4 and S5). The AEC-c matrices also showed good replication across all frequency bands for both groups (*r_s_* > 0.67; range: 0.68–0.88; Supplementary Fig. S2). The PLI matrices exhibited more variability, particularly in the theta band (Supplementary Fig. S3). This was reflected by much lower correlations between the matrices from cohort 1 and cohort 2 (*r_s_* > 0.08; range: 0.09-0.41). These correlations were, however, still significant (*p* < .001).

### 3.5 Classification of AD patients and control subjects based on whole-brain functional connectivity values

**Fig. 6.**
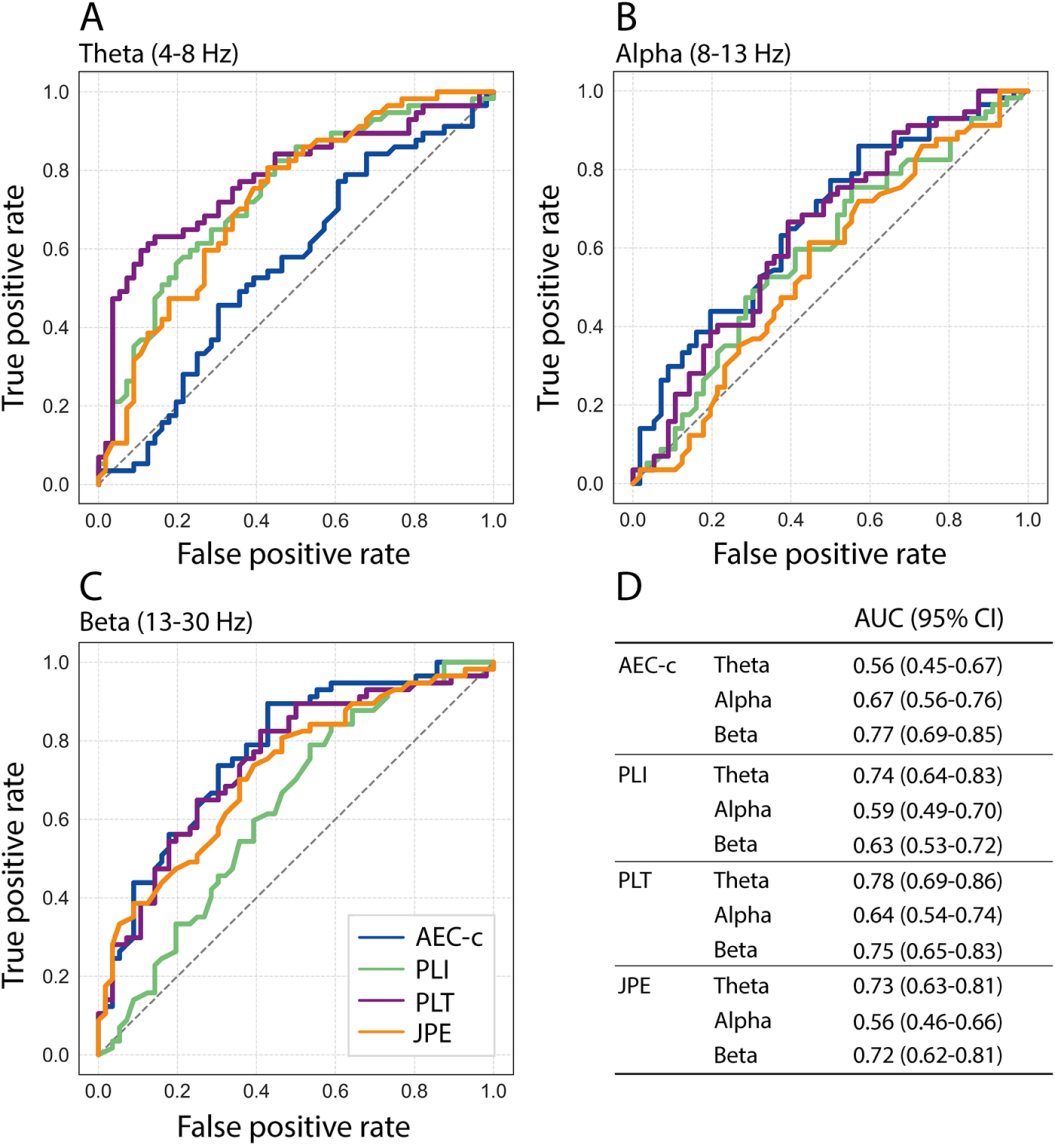
ROC curves for classification of AD patients and control subjects based on whole-brain functional connectivity. Classification based on whole-brain AEC-c, PLI, PLT, or JPE values in the theta (A), alpha (B), and beta (C) band. (D) Corresponding Area Under the Curve (AUC) values and 95% confidence intervals (CI).

Figure 6 shows the ROC curves for the classification of AD patients and control subjects based on whole-brain AEC-c, PLI, PLT, or JPE values in the theta (Fig. 6A), alpha (Fig. 6B), and beta bands (Fig. 6C). Corresponding AUCs and 95% confidence intervals are shown in Figure 6D.

For the AEC-c, the highest AUC was obtained for the beta band (0.77), while for the PLI (0.74), PLT (0.78), and JPE (0.73), the highest AUC values were achieved for the theta band. Overall, lowest classification performance was achieved based on functional connectivity in the alpha band. The classification performance of whole-brain AEC-c beta, PLI theta, PLT theta and beta, and JPE theta and beta was similar, with AUC values ranging from 0.72 to 0.78.

### 3.6 The relationship between whole-brain functional connectivity and coupling strength in a whole-brain computational model

**Fig. 7.**
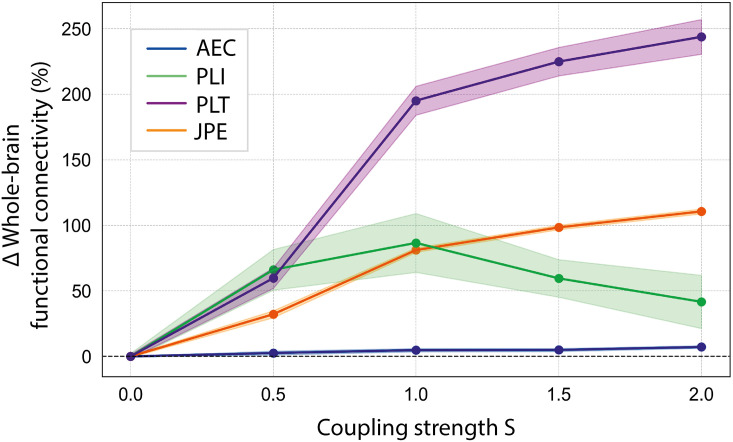
Whole-brain functional connectivity as a function of coupling strength. Displayed are mean (±SD) percentage changes in whole-brain functional connectivity (estimated by AEC, PLI, PLT, and JPE) derived from simulated MEG data across varying values of coupling strength S in the model. Percentage changes were computed relative to the functional connectivity values at coupling strength *S* = 0, to ensure a consistent scale across measures.

Figure 7 shows the percentage change in whole-brain functional connectivity (estimated by AEC, PLT, PLI, and JPE), derived from simulated MEG data, as a function of the coupling strength (*S*) between the neural mass models or “regions” in a whole-brain computational model. Percentage changes were computed relative to the functional connectivity values at coupling strength *S* = 0, to ensure a consistent scale across measures. The individual scales for each measure are shown in Supplementary Figure S8. The AEC, PLT, and JPE all followed a similar trend, with functional connectivity increasing as the coupling strength rose. The percentage change in whole-brain functional connectivity was largest for the PLT, followed by the JPE. Both measures exhibited the steepest increase between *S* = 0.5 and *S* = 1.0, followed by a more gradual slope from *S* = 1.0 to S = 2.0. The AEC showed a consistent increase with coupling strength but with a percentage change smaller than that of the PLT and JPE. In contrast, the PLI displayed a distinct pattern, rising significantly up to *S* = 1.0 before decreasing at higher coupling strengths, forming an inverted U-shaped curve. Additionally, the PLI exhibited the largest standard deviation among the measures, reflecting greater variability.

## 4 Discussion

Our study demonstrates that the PLT and JPE can reliably detect neurophysiological abnormalities related to AD from resting-state MEG recordings. Across two independent cohorts, the novel measures identified consistent whole-brain and regional group differences in functional connectivity between patients with dementia due to AD and control subjects, particularly in the theta and beta bands. Their functional connectivity matrices moreover exhibited strong within-group consistency across cohorts for both AD patients and control subjects in the theta, alpha, and beta bands. The PLT and JPE were shown to be sensitive to changes in functional coupling strength in a whole-brain computational model. Overall, the novel measures, which capture rapid and transient changes in functional connectivity, performed similarly or better than the AEC-c and PLI, two previously validated measures that capture functional connectivity over slower timescales.

### 4.1 Group differences in functional connectivity across cohorts

Whole-brain differences in functional connectivity between AD patients and control subjects could be replicated across cohorts for all measures under investigation (AEC-c, PLI, PLT, and JPE), although not in each frequency band. Group differences varied between measures both in terms of which frequency band(s) exhibited significant group differences and the direction of the effect. This variability between measures aligns with the idea that the brain employs multiple modes of cortical communication that may operate at different temporal scales simultaneously (Hindriks & Tewarie, 2023; Siegel et al., 2012; Siems & Siegel, 2020). Combining results from multiple functional connectivity measures may, therefore, provide a more comprehensive and nuanced understanding of brain dynamics than relying on a single measure. Across cohorts, AEC-c beta (lower in AD patients than in control subjects), PLT theta (higher in AD patients), and JPE theta (lower in AD patients) showed the most pronounced group differences at both whole-brain and regional level. This is consistent with previous research demonstrating that the AEC-c is a reliable metric for connectivity estimation (Colclough et al., 2016) that can effectively replicate group differences between AD patients and control subjects based on EEG and MEG recordings (Briels et al., 2020; Schoonhoven et al., 2022). We have demonstrated that the PLT and JPE can identify significant and replicable differences in functional connectivity between patients with dementia due to AD and control subjects based on MEG recordings. This complements previous work indicating that the PLT and JPE are highly effective in detecting neurophysiological abnormalities, particularly neuronal hyperexcitability, in early stage AD (Scheijbeler et al., 2022, 2025; Stam & de Haan, 2024). While a difference in whole-brain PLI theta could be replicated across cohorts, it was challenging to replicate differences in functional connectivity estimated by PLI on a regional level. This will be discussed in more detail in the following section.

The spatial distribution of functional connectivity differences between AD patients and control subjects was similar across cohorts, as reflected by the significant correlations between the regional difference scores from each cohort. We observed significant correlations across all frequency bands for the AEC-c, PLT, and JPE, whereas for the PLI, significant correlations were restricted to the alpha band. Alpha oscillations are characterized by a relatively high amplitude, resulting in a higher signal-to-noise ratio in MEG recordings (Martin-Buro et al., 2016). This may make it easier to detect meaningful differences and could explain why the strongest overall correlations were observed in this frequency range. For AEC-c theta, PLI alpha, PLT alpha, and JPE alpha, we observed relatively high correlations (*r* = 0.56–0.82, *p* < .001) between the regional difference scores from each cohort, while only a few regions showed significant and replicable group differences. This may indicate that the measures are sensitive to more subtle, widespread differences in functional connectivity within these frequency bands.

### 4.2 Within-group functional connectivity across cohorts

The PLT and JPE outperformed the AEC-c and PLI in replicating within-group functional connectivity patterns across cohorts. Within-group functional connectivity matrices were highly consistent for both AD patients and control subjects in the theta, alpha, and beta bands. AEC-c matrices also exhibited good within-group consistency for both AD patients and control subjects across all frequency bands. PLI matrices showed highest variability, particularly in the theta band, leading to relatively low—though still significant—within-group correlations between the connectivity matrices from cohort 1 and cohort 2. The PLI appears to be a less robust estimator than the other functional connectivity measures, resulting in a reduced ability to extract consistent functional connectivity patterns from noisy electrophysiological recordings. While this has been suggested previously (Colclough et al., 2016), another study did report good test–retest reliability for the PLI (Hardmeier et al., 2014). The noisier and less consistent PLI estimates within groups may have obscured subtle connectivity differences between groups, hindering the detection and replication of regional group differences. For the PLI, PLT, and JPE, highest within-group correlations were found in the alpha band, both for AD patients and control subjects. For the AEC-c, the highest correlation was observed in the alpha band for control subjects, but in the beta band for AD patients. In AD, the amplitude of the posterior dominant rhythm is known to decrease, and oscillatory slowing occurs (de Haan et al., 2008; Engels et al., 2016; Garcés et al., 2013; Jeong, 2004). This may explain the increased variability in amplitude-based functional connectivity observed in the alpha band in this patient group. Qualitative visual inspection of the group-level functional connectivity matrices (Supplementary Figs. S2–S5), furthermore, suggests a distinction between the topographies derived from the novel measures (PLT and JPE) and those based on conventional measures (PLI and AEC-c). For PLI and AEC-c, the most pronounced connectivity tends to occur within relatively small subsets of brain regions, which appear visually as bright, localized blocks in the symmetric connectivity matrices. In contrast, for PLT, and especially for JPE, certain regions exhibit strong connectivity to nearly all other regions, producing bright rows and columns in the matrices. This suggests that the novel measures may more readily capture “hub-like” regions that connect broadly across the brain, rather than localized modular networks.

### 4.3 Classification of individuals

Simple logistic regression models achieved AUC values ranging from 0.72 to 0.78 for the classification of AD patients and control subjects based on whole-brain AEC-c beta, PLI theta, PLT theta or beta, or JPE theta or beta values. While more advanced classification methods may have yielded higher AUC values, as has been demonstrated in previous studies using AEC-c or PLI values to classify AD patients and control subjects (Ajra et al., 2023; Klepl et al., 2022; Nobukawa et al., 2020; Shan et al., 2022), the primary aim of this analysis was to compare the classification performance of the novel measures with that of the AEC-c and PLI. The results suggest that the PLT and JPE perform at least as well, if not slightly better, than the AEC-c and PLI in this classification task, further supporting the potential of the novel measures. In a previous study, we showed that JPE theta achieved an AUC of 0.78 for classifying patients with early stage AD and control subjects (Scheijbeler et al., 2022). Given the more advanced disease stage, the current AD versus controls classification was expected to be less challenging. We do, however, not report a higher AUC. Previous research has highlighted the sensitivity of the JPE to early AD-related neurophysiological changes, such as neuronal hyperexcitability (Scheijbeler et al., 2022, 2025; Stam & de Haan, 2024; van Nifterick et al., 2023). It is possible that the JPE follows a non-linear trajectory throughout the course of AD, and that it is particularly sensitive to changes in early stages. Future research should investigate whether JPE results are also replicable in early stages of AD and whether group differences are indeed more pronounced during this stage.

### 4.4 Construct validity of the functional connectivity measures

Finally, we evaluated the effect of systematically increasing the functional coupling strength between neural mass models or “regions” in a whole-brain computational model on functional connectivity. The AEC, PLT, and JPE all followed a similar trend: functional connectivity increased as the coupling strength increased. The percentage change in whole-brain functional connectivity was largest for the PLT, followed by the JPE and then AEC. The PLI followed an inverted U-shaped curve; functional connectivity increased until *S* = 1.0, after which connectivity decreased.

The “critical brain hypothesis” poses that the brain operates near a critical phase transition, where there is a balance between excitation and inhibition and information processing is optimized (Beggs, 2008; Chialvo, 2010; Cocchi et al., 2017; Shew et al., 2011; Shew & Plenz, 2013). By systematically increasing the coupling strength from S = 0 to S = 2.0, the level of excitatory interaction between “regions” in the model was increased. This procedure was hypothesized to drive the system closer to its critical point. It is hypothesized that near this point, regions can effectively synchronize and communicate, leading to increased functional connectivity (Avramiea et al., 2022; Fuscà et al., 2023; Kitzbichler et al., 2009; Stam et al., 2023). Beyond this point, further increases in coupling may push the system into a state of intrinsic overactivity or saturation. In this state, regions may become less responsive to external inputs due to high local activity, resulting in functional decoupling. This may explain why the PLT and JPE showed the strongest increase at moderate coupling strengths, with a more gradual rise at higher strengths, and why the PLI even exhibited a decline in functional connectivity at high coupling strengths. Previous studies, both experimental and computational, have found that functional connectivity values reach their maximum in systems that are optimally balanced (Avramiea et al., 2022; Fuscà et al., 2023; Stam et al., 2023). The AEC increased monotonically with coupling strength, perhaps because amplitude-based measures are less sensitive to the complex temporal and phase relationships that characterize critical dynamics. The fact that the PLT does not exhibit an inverted U-shape suggests that the PLI and PLT, despite both involving phase information, capture different aspects of the relationship between oscillatory signals. At high coupling strengths, high intrinsic activity may amplify transient leading-lagging roles, even if long-term phase synchronization is disrupted. Our results support the construct validity of the novel functional connectivity measures by demonstrating their ability to capture meaningful changes in coupling strength, and show that, within a computational model, they are more sensitive to these changes than the established AEC and PLI.

### 4.5 Limitations

Our study has some potential limitations that are important to keep in mind. First, all control subjects included in this study had SCD. Although the majority of these subjects were confirmed amyloid negative, and, therefore, not preclinical AD patients, they may still have exhibited subtle neural changes, which could contribute to variability in the observed results and should, therefore, be considered when interpreting group differences. In addition, as given in Table 2, control subjects were significantly younger than the AD patients in both cohorts, which may have affected the observed group differences. In Supplementary Figure S6, we present the Pearson correlation between whole-brain functional connectivity and age. Only PLT beta showed a significant correlation with age in AD patients (*r* = 0.36, *p* < .01). None of the other measures showed a significant correlation with age. This suggests that the effect of age on our results was small. Second, as shown in Supplementary Figure S7, we observed significant correlations between whole-brain functional connectivity and whole-brain relative power in the corresponding frequency band, in both AD patients and control subjects. While we have demonstrated that the functional connectivity measures are sensitive to changes in coupling strength in a whole-brain computational model, it is important to recognize that the measures are not independent from (relative) power, either being affected directly, or indirectly through the signal-to-noise ratio of the data (Muthukumaraswamy & Singh, 2011). Functional connectivity and (relative) power measures may, therefore, convey partially redundant information. Third, we performed pairwise orthogonalization (Hipp et al., 2012) prior to AEC estimation to reduce the effects of field spread. While this procedure mitigates the effects of linear signal mixing between each pair of sources, it does not account for cases in which three or more sources are interconnected at zero lag (J. M. Palva et al., 2018). To address residual field spread, some studies have proposed the use of multivariate orthogonalization (Colclough et al., 2015; Soto et al., 2016). However, because this procedure is highly stringent and can remove a substantial portion of the underlying neural signal, we did not employ it in the present study. Finally, it is important to note that entropy measures such as the JPE are sensitive to the timescale at which they are evaluated (Costa et al., 2002, 2005; Kosciessa et al., 2020; Scheijbeler et al., 2022; van Nifterick et al., 2023; Yin et al., 2019). Factors such as time lag *τ* and the frequency band used for signal filtering influence the timescale being analyzed and, consequently, the entropy results (Scheijbeler et al., 2022; van Nifterick et al., 2023). This study presents JPE results computed with a time lag *τ* of 1 in the theta band, as these settings yielded significant results in previous research (Scheijbeler et al., 2022, 2025). Our results suggest that this timescale is sensitive in the context of AD and that JPE findings can be replicated at this scale. This does, however, not imply that it is the only relevant timescale for MEG analysis. Future research should examine whether the performance of the JPE can be optimized further with alternative parameter settings. Moreover, it should be investigated whether the PLT and/or JPE can produce meaningful results in other neurological or psychiatric conditions.

## 5 Conclusion

We have demonstrated that the PLT and JPE, when applied to eyes-closed resting-state MEG recordings from two independent cohorts of AD patients and control subjects, can identify consistent patterns of between- and within-group functional connectivity. Our findings show that the measures outperform the PLI and, to a lesser extent, the AEC-c, in terms of sensitivity to group differences and replicability across cohorts. Additionally, the novel measures effectively capture meaningful changes in coupling strength, as demonstrated using a whole-brain computational model. Since accumulating evidence identifies neuronal hyperexcitability as a key pathological process in early AD and a potential therapeutic target, coupled with the fact that the PLT and JPE have previously been demonstrated to be sensitive to this phenomenon, the novel functional connectivity measures could play a valuable role in early disease detection and the evaluation of treatments aimed at restoring E-I balance in AD.

## Supplementary Material

Supplementary Material

## Data Availability

Due to informed consent restrictions and applicable data protection regulations (including GDPR), the data underlying this study cannot be made publicly available. Data may be obtained from the corresponding author upon reasonable request, subject to a formal data sharing agreement. The analyses underlying this study’s findings, including time series analyses (discrete FFT, Hilbert transform, and functional connectivity estimation) and MEG data simulation, were conducted using BrainWave software (version 0.9.136.26, available at https://github.com/CornelisStam/BrainWave).
