# Supplementary material for: Replication and validation of two novel magnetoencephalography functional connectivity measures in Alzheimer’s disease
Source: Imaging Neurosci (Camb). 2026 Jan 27;4:IMAG.a.1113. doi: 10.1162/IMAG.a.1113 (PMC12849232; doi:10.1162/IMAG.a.1113)
Supplement: Supplementary Material [file IMAG.a.1113_supp.pdf]

Supplementary Materials

Supplementary Table 1 Subdivision of parcels as included in the automated anatomical labeling (AAL) atlas. Presented parcel numbers (#) are based on Gong et al. (2009).

**Parcel #**		**Parcel #**	
**Frontal (Left/Right)**		**Occipital (Left/Right)**	
1/40	Rectus	22/61	Occipital_Sup
2/41	Olfactory	23/62	Occipital_Mid
3/42	Frontal_sup_Orb	24/63	Occipital_Inf
4/43	Frontal_med_Orb	25/64	Calcarine
5/44	Frontal_Mid_Orb	26/65	Cuneus
6/45	Frontal_Inf_Orb	27/66	Lingual
7/46	Frontal_Sup		
8/47	Frontal_Mid	**Temporal (Left/Right)**	
9/48	Frontal_Inf_Oper	28/67	Fusiform
10/49	Frontal_Inf_Tri	29/68	Heschl
11/50	Frontal_Sup_Medial	30/69	Temporal_Sup
12/51	Supp_Motor_Area	31/70	Temporal_Mid
13/52	Paracentral_Lobule	32/71	Temporal_Inf
14/53	Precentral	33/72	Temporal_Pole_Sup
15/54	Rolandic_Oper	34/73	Temporal_Pole_Mid
		35/74	ParaHippocampal
**Parietal (Left/Right)**		36/75	Cingulum_Ant
16/55	Postcentral	37/76	Cingulum_Mid
17/56	Parietal_Sup	38/77	Cingulum_Post
18/57	Parietal_Inf	39/78	Insula
19/58	SupraMarginal		
20/59	Angular	**Subcortical (Left/Right)**	
21/60	Precuneus	79/80	Hippocampus

Supplementary Table 2 Technical definitions of the functional connectivity measures under investigation.

Measure	Equation	Source
Amplitude envelope[a]	$A_t = \sqrt{X_t^{\,2} + Y_t^{\,2}}$	
Phase Lag Index	$PLI_{i,j} = \mid sign\left[sin\left(\varphi_{i,j}\right)\right] \mid$	Stam et al. (2007)
Phase Lag Time	$PLT_{i,j} = 1 - e^{T}$	Stam and de Haan (2024)
Joint Permutation Entropy[b]	$JPE_{inv} = -\frac{H(n)}{log(n! * n! - 2n! + 1)}$	Scheijbeler et al. (2022)
**Symbol definitions**		
X_t = Original signal at time point *t*		
Y_t = Hilbert transform of the original signal at time point *t*		
$\varphi_{i,j}$ = Instantaneous phase difference between signals *i* and *j*		
T = Average duration (in seconds) of an interval between two successive sign changes of the phase difference.		
H = Shannon entropy		
n = Ordinal pattern length or embedding dimension		

[a.] The amplitude envelope correlation (AEC) is defined as the Pearson correlation of the amplitude envelopes of pairs of time series (Brookes et al., 2011; Bruns et al., 2000; Hipp et al., 2012). Prior to computation of the AEC, pair-wise orthogonalization of the data was performed to correct for volume conduction (Brookes et al., 2012).

[b.] For simplicity, we assume the sign reversal, and thus throughout the paper, "JPE" is used to denote "JPE_{inv}".

Supplementary Table 3 Whole-brain functional connectivity differences between AD patients and control subjects. The table displays the mean whole-brain functional connectivity values along with their standard deviations. Significant group differences are highlighted. *$p < .05$; ** $p < .01$; *** $p < .001$.

	**Cohort 1 (*n* = 57)**			**Cohort 2 (*n* = 56)**		
	**AD (*n* = 28)**	**Controls (*n* = 29)**	**P-value**	**AD (*n* = 29)**	**Controls (*n* = 27)**	**P-value**
AEC-c (mean ± SD)						
Theta	0.519 (0.012)	0.519 (0.014)	0.80	0.519 (0.013)	0.516 (0.012)	0.35
Alpha	0.520 (0.013)	0.530 (0.018)	**0.029***	0.519 (0.012)	0.529 (0.017)	**0.026***
Beta	0.513 (0.006)	0.524 (0.016)	**0.001****	0.513 (0.007)	0.521 (0.009)	**0.002****
PLI (mean ± SD)						
Theta	0.101 (0.004)	0.098 (0.004)	**0.016***	0.102 (0.006)	0.097 (0.003)	**0.002****
Alpha	0.096 (0.008)	0.096 (0.008)	0.89	0.094 (0.007)	0.100 (0.009)	**0.012***
Beta	0.051 (0.002)	0.053 (0.003)	**0.026***	0.052 (0.002)	0.053 (0.003)	0.10
PLT (mean ± SD)						
Theta	0.223 (0.010)	0.214 (0.008)	**0.001****	0.224 (0.011)	0.214 (0.007)	**<0.001*****
Alpha	0.189 (0.010)	0.192 (0.012)	0.29	0.189 (0.008)	0.197 (0.010)	**0.004****
Beta	0.062 (0.002)	0.064 (0.003)	**0.001****	0.063 (0.003)	0.064 (0.002)	**0.017***
JPE (mean ± SD)						
Theta	0.391 (0.004)	0.395 (0.005)	**0.005****	0.390 (0.002)	0.393 (0.004)	**0.001****
Alpha	0.375 (0.004)	0.375 (0.004)	0.988	0.375 (0.003)	0.377 (0.004)	0.10
Beta	0.356 (0.002)	0.357 (0.002)	**0.007****	0.356 (0.002)	0.358 (0.002)	**0.006****

A

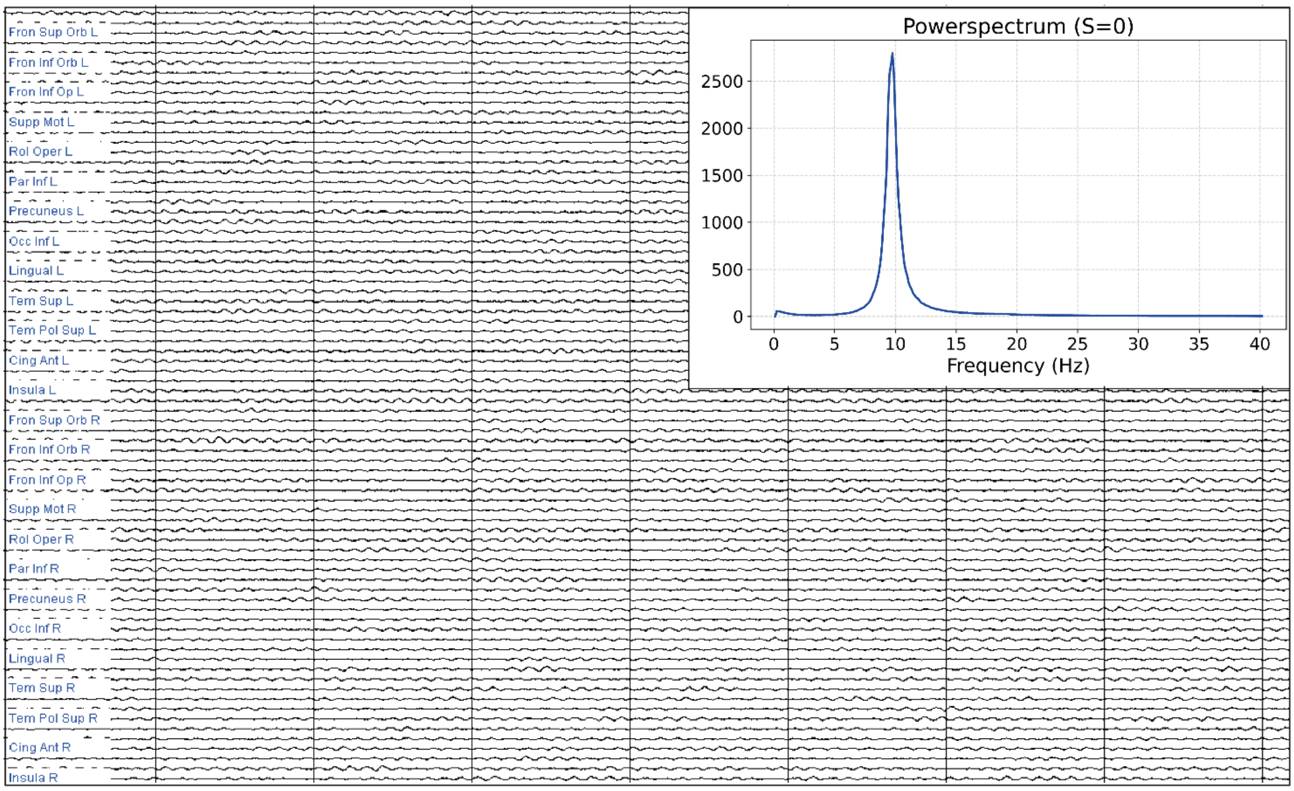

Fron Sup Orb L
Fron Inf Orb L
Fron Inf Op L
Supp Mot L
Rol Oper L
Par Inf L
Precuneus L
Occ Inf L
Lingual L
Tem Sup L
Tem Pol Sup L
Cing Ant L
Insula L
Fron Sup Orb R
Fron Inf Orb R
Fron Inf Op R
Supp Mot R
Rol Oper R
Par Inf R
Precuneus R
Occ Inf R
Lingual R
Tem Sup R
Tem Pol Sup R
Cing Ant R
Insula R
Powerspectrum (S=0)
2500
2000
1500
1000
500
0
0
5
10
15
20
25
30
35
40
Frequency (Hz)

B

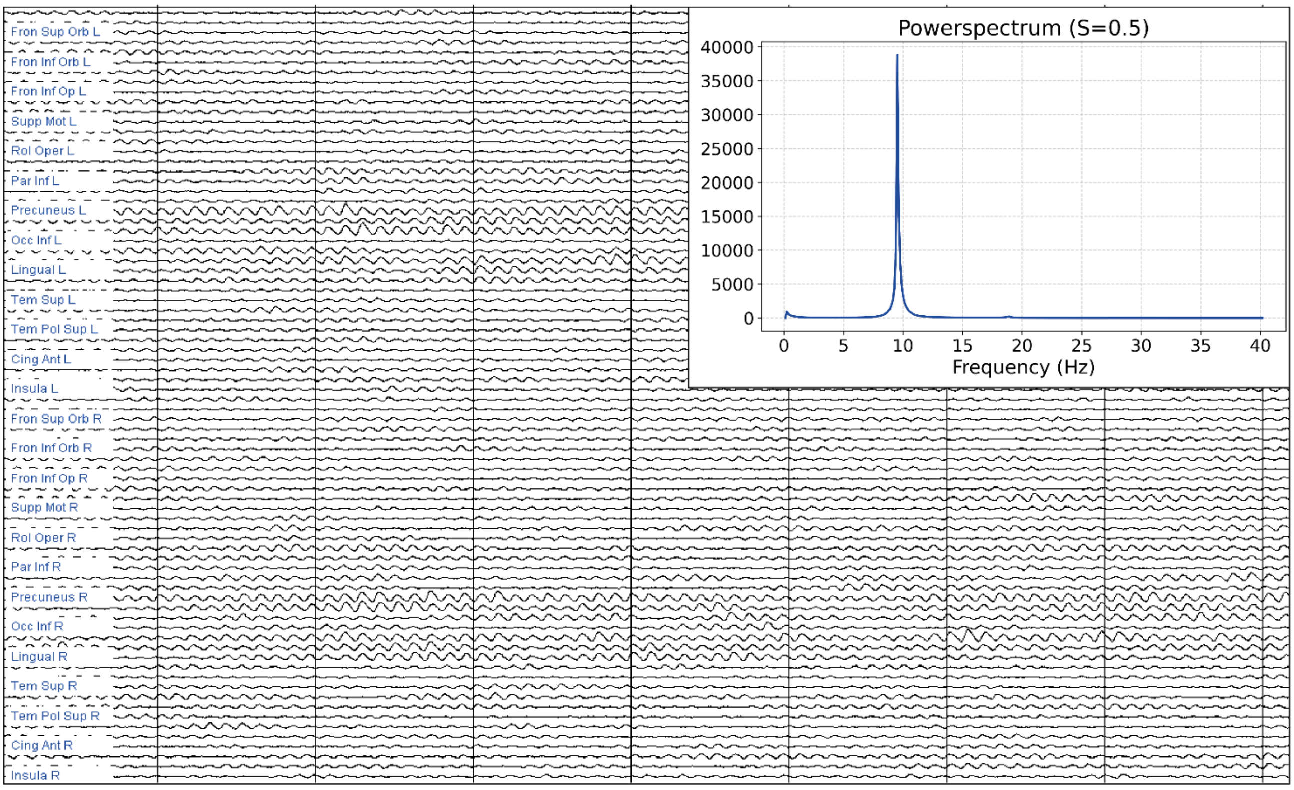

Fron Sup Orb L
Fron Inf Orb L
Fron Inf Op L
Supp Mot L
Rol Oper L
Par Inf L
Precuneus L
Occ Inf L
Lingual L
Tem Sup L
Tem Pol Sup L
Cing Ant L
Insula L
Fron Sup Orb R
Fron Inf Orb R
Fron Inf Op R
Supp Mot R
Rol Oper R
Par Inf R
Precuneus R
Occ Inf R
Lingual R
Tem Sup R
Tem Pol Sup R
Cing Ant R
Insula R
Powerspectrum (S=0.5)
40000
35000
30000
25000
20000
15000
10000
5000
0
0
5
10
15
20
25
30
35
40
Frequency (Hz)

C

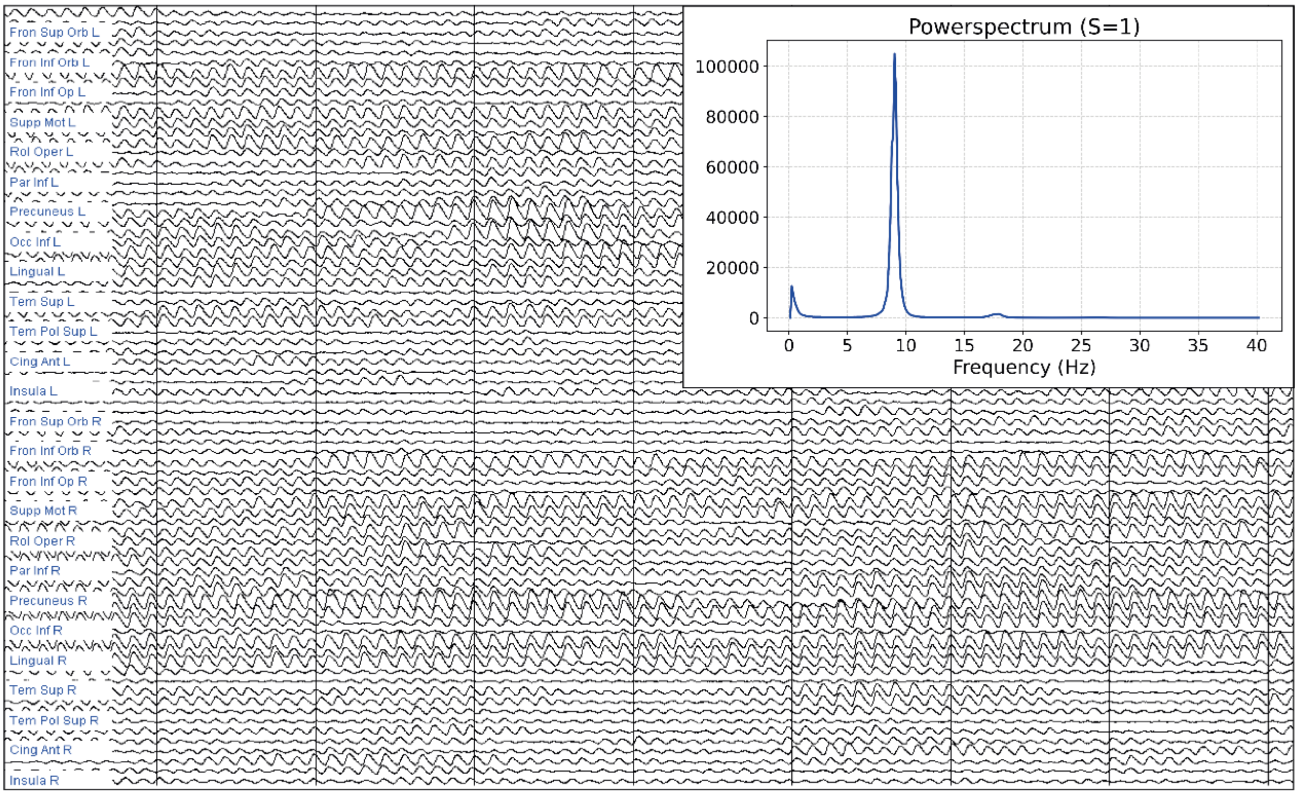
Fron Sup Orb L
Fron Inf Orb L
Fron Inf Op L
Supp Mot L
Rol Oper L
Par Inf L
Precuneus L
Occ Inf L
Lingual L
Tem Sup L
Tem Pol Sup L
Cing Ant L
Insula L
Fron Sup Orb R
Fron Inf Orb R
Fron Inf Op R
Supp Mot R
Rol Oper R
Par Inf R
Precuneus R
Occ Inf R
Lingual R
Tem Sup R
Tem Pol Sup R
Cing Ant R
Insula R
Powerspectrum (S=1)
100000
80000
60000
40000
20000
0
0 5 10 15 20 25 30 35 40
Frequency (Hz)

D

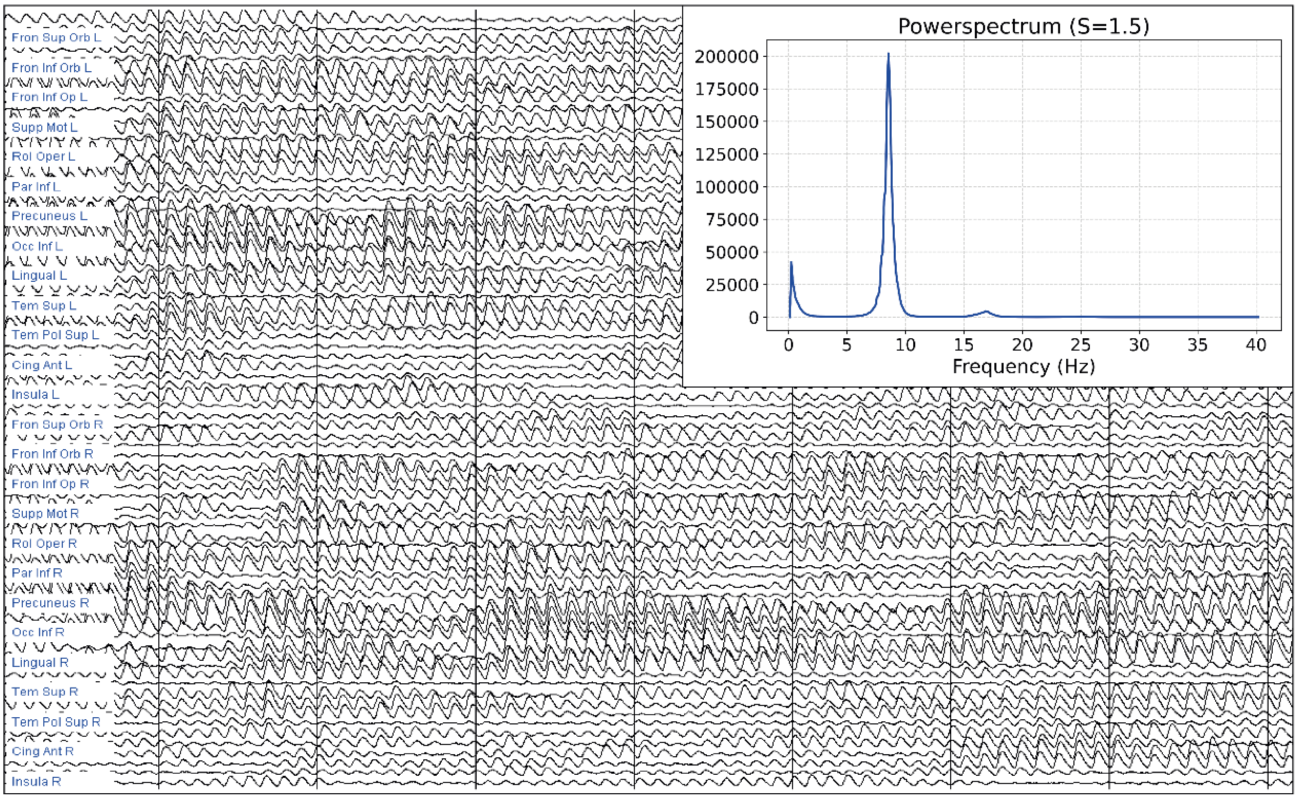
Fron Sup Orb L
Fron Inf Orb L
Fron Inf Op L
Supp Mot L
Rol Oper L
Par Inf L
Precuneus L
Occ Inf L
Lingual L
Tem Sup L
Tem Pol Sup L
Cing Ant L
Insula L
Fron Sup Orb R
Fron Inf Orb R
Fron Inf Op R
Supp Mot R
Rol Oper R
Par Inf R
Precuneus R
Occ Inf R
Lingual R
Tem Sup R
Tem Pol Sup R
Cing Ant R
Insula R
Powerspectrum (S=1.5)
200000
175000
150000
125000
100000
75000
50000
25000
0
0 5 10 15 20 25 30 35 40
Frequency (Hz)

E

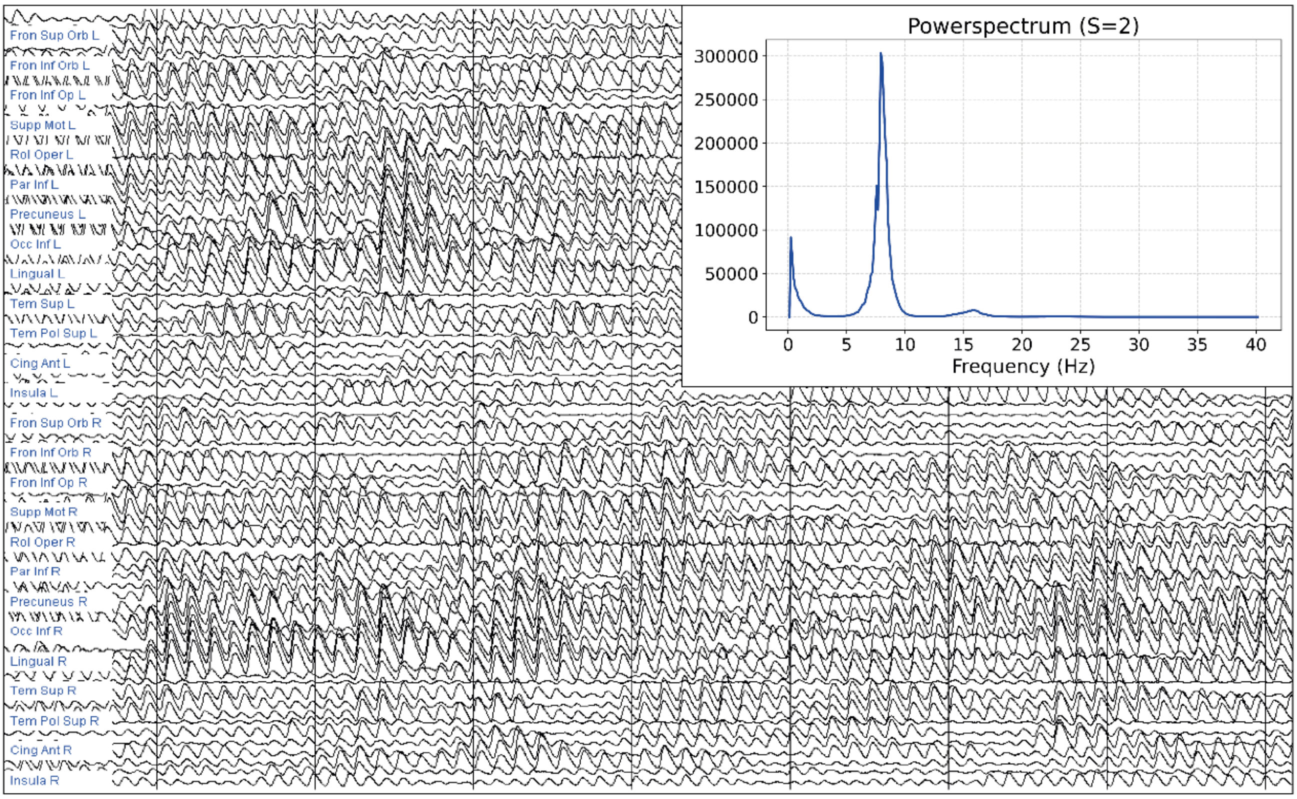

Supplementary Figure 1 Simulated MEG signals and corresponding whole-brain average power spectra at varying levels of coupling. Simulated MEG signals and their corresponding whole-brain average power spectra (power shown in arbitrary units) are shown for different levels of global coupling strength *S*. **(A)** *S* = 0 (no coupling), **(B)** *S* = 0.5, **(C)** *S* = 1, **(D)** *S* = 1.5, and **(E)** *S* = 2. Each horizontal line represents a simulated signal for one of 78 parcels (as included in the AAL atlas, see Supplementary Table 1). Vertical black lines indicate timestamps of 1 second. The figure illustrates how increasing coupling strength affects the dynamics and spectral properties of the simulated signals.

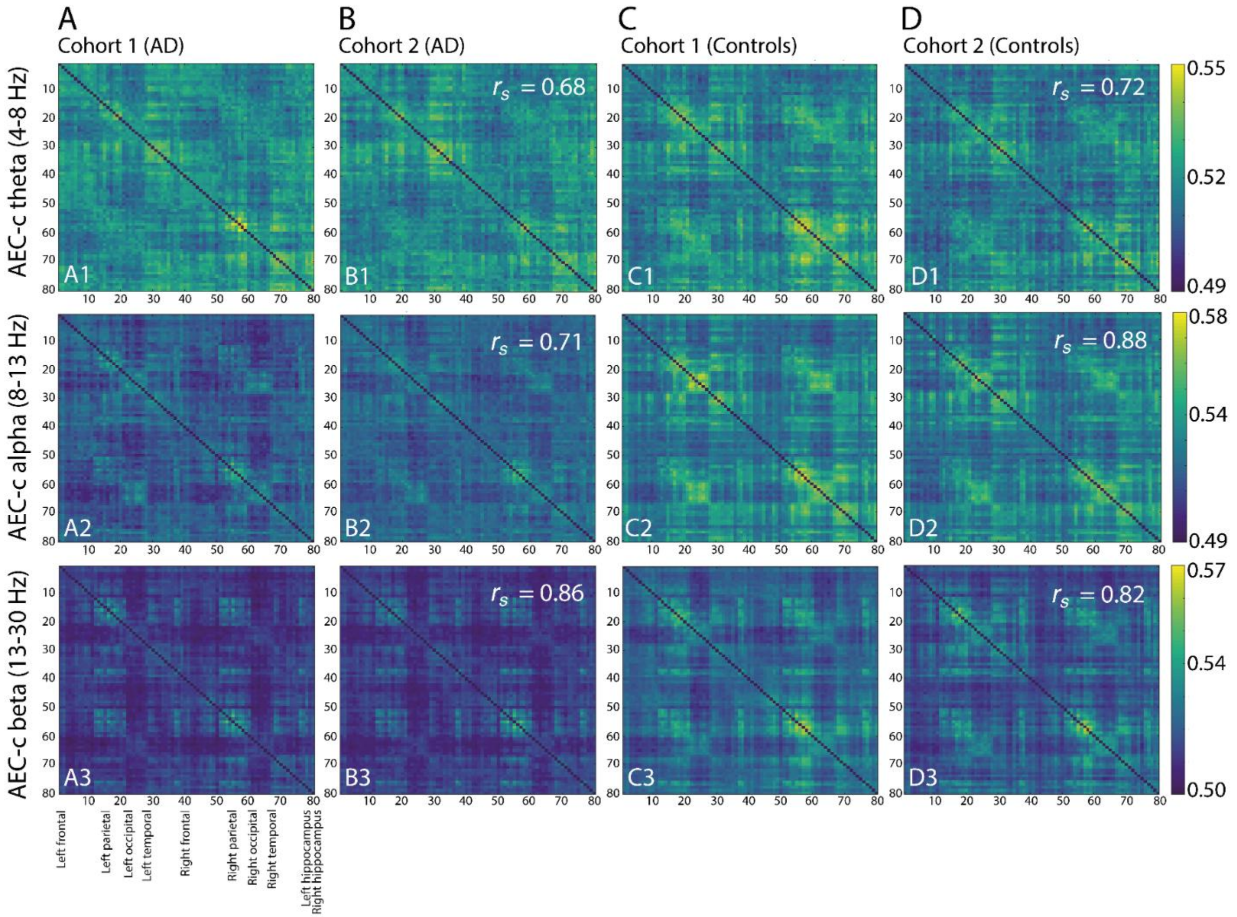

Supplementary Figure 2 Average AEC-c functional connectivity matrices of AD patients and control subjects in two independent cohorts. (A1-A3) Average AEC-c matrices of AD patients in cohort 1. **(B1-B3)** Average AEC-c matrices of AD patients in cohort 2. The Spearman correlation coefficients in the top right corner of each matrix quantify the consistency of connectivity patterns between cohort 1 and cohort 2 for the AD patient group. **(C1-C3)** Average AEC-c matrices of control subjects in cohort 1. **(D1-D3)** Average AEC-c matrices of control subjects in cohort 2. The Spearman correlation coefficients in the top right corner of each matrix quantify the consistency of connectivity patterns between cohort 1 and cohort 2 for the control group. Matrices are presented for the theta (4-8 Hz), alpha (8-13 Hz) and beta (13-30 Hz) frequency bands. Regions of interest (1-80) are ordered from the left to the right hemisphere (see Supplementary Table 1). All Spearman correlations had a significance level of $p < .001$ (FDR-corrected).

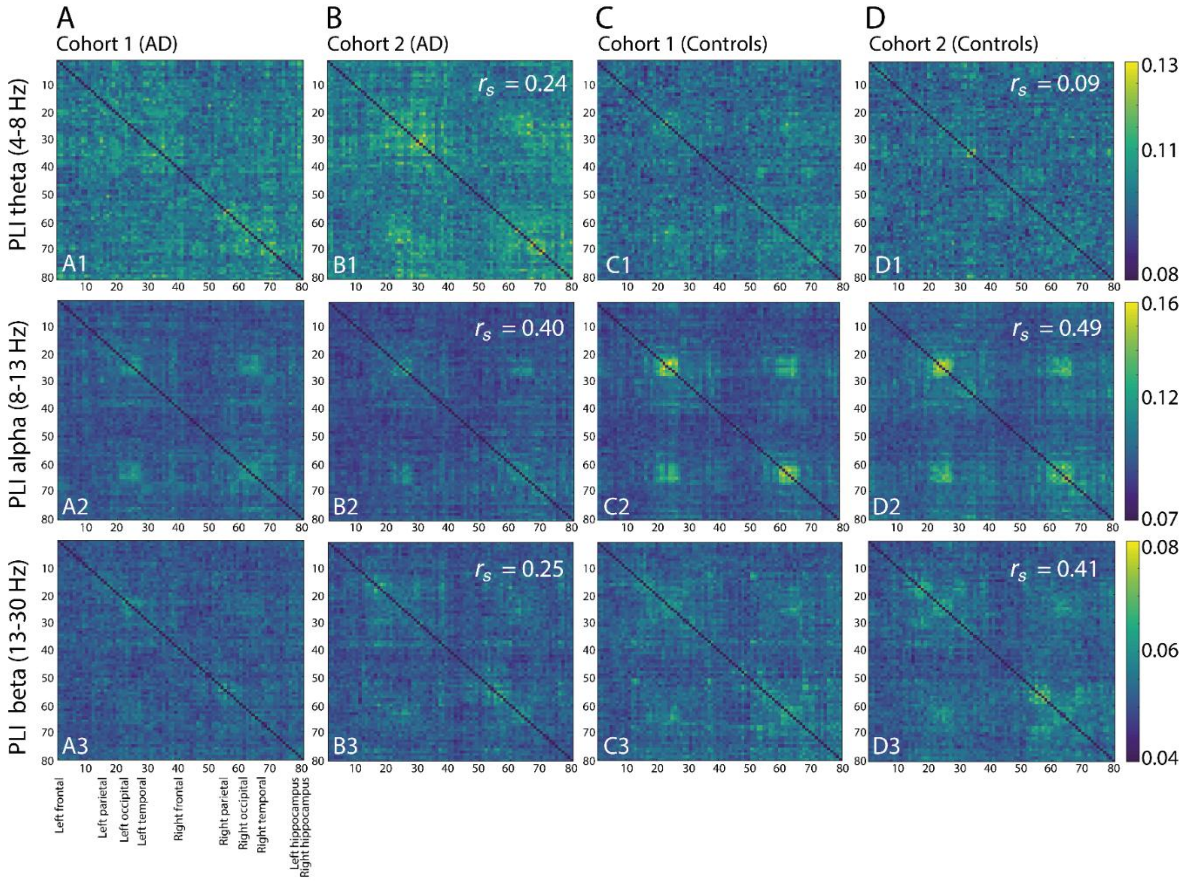

Supplementary Figure 3 Average PLI functional connectivity matrices of AD patients and control subjects in two independent cohorts. (A1-A3) Average PLI matrices of AD patients in cohort 1. **(B1-B3)** Average PLI matrices of AD patients in cohort 2. The Spearman correlation coefficients in the top right corner of each matrix quantify the consistency of connectivity patterns between cohort 1 and cohort 2 for the AD patient group. **(C1-C3)** Average PLI matrices of control subjects in cohort 1. **(D1-D3)** Average PLI matrices of control subjects in cohort 2. The Spearman correlation coefficients in the top right corner of each matrix quantify the consistency of connectivity patterns between cohort 1 and cohort 2 for the control group. Matrices are presented for the theta (4-8 Hz), alpha (8-13 Hz) and beta (13-30 Hz) frequency bands. Regions of interest (1-80) are ordered from the left to the right hemisphere (see Supplementary Table 1). All Spearman correlations had a significance level of $p < .001$ (FDR-corrected).

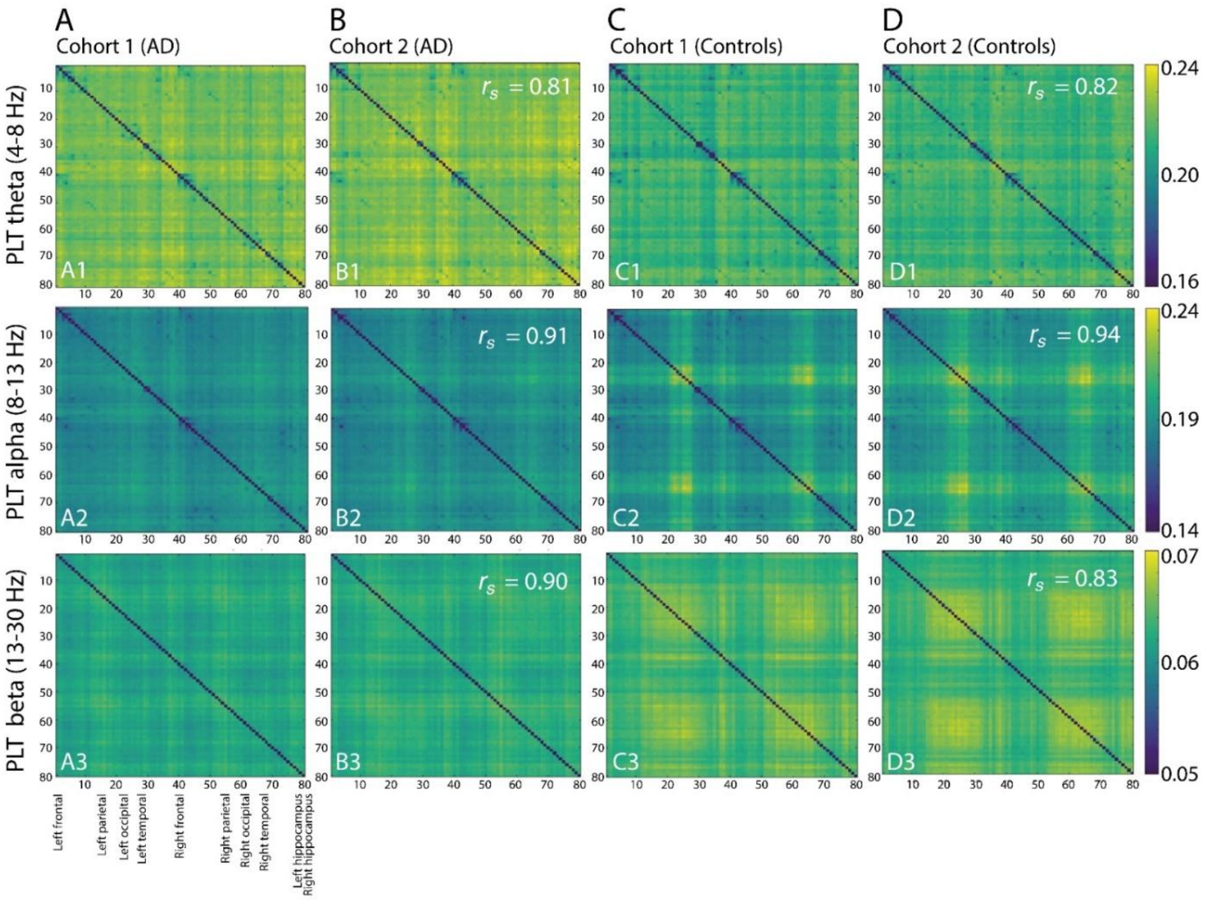

Supplementary Figure 4 Average PLT functional connectivity matrices of AD patients and control subjects in two independent cohorts. (A1-A3) Average PLT matrices of AD patients in cohort 1. **(B1-B3)** Average PLT matrices of AD patients in cohort 2. The Spearman correlation coefficients in the top right corner of each matrix quantify the consistency of connectivity patterns between cohort 1 and cohort 2 for the AD patient group. **(C1-C3)** Average PLT matrices of control subjects in cohort 1. **(D1-D3)** Average PLT matrices of control subjects in cohort 2. The Spearman correlation coefficients in the top right corner of each matrix quantify the consistency of connectivity patterns between cohort 1 and cohort 2 for the control group. Matrices are presented for the theta (4-8 Hz), alpha (8-13 Hz) and beta (13-30 Hz) frequency bands. Regions of interest (1-80) are ordered from the left to the right hemisphere (see Supplementary Table 1). All Spearman correlations had a significance level of $p < .001$ (FDR-corrected).

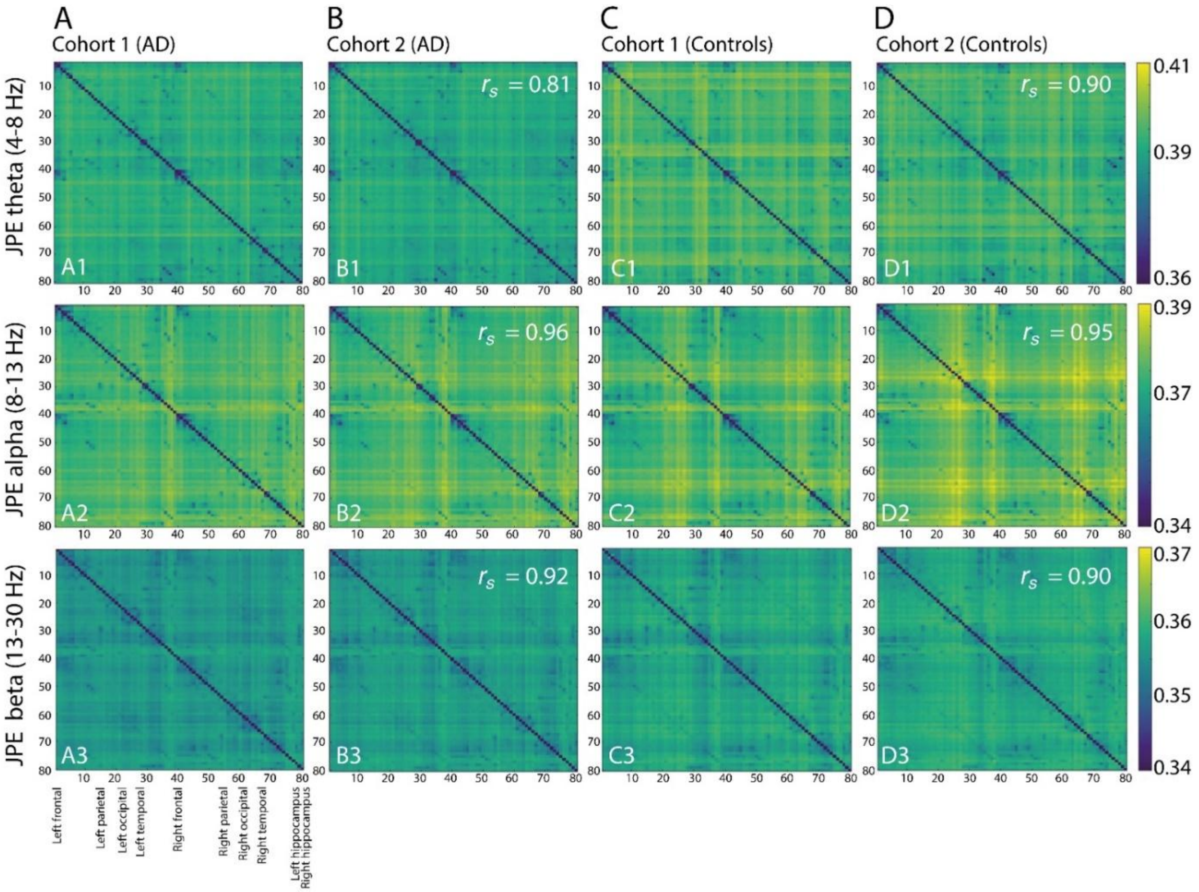

Supplementary Figure 5 Average JPE functional connectivity matrices of AD patients and control subjects in two independent cohorts. (A1-A3) Average JPE matrices of AD patients in cohort 1. **(B1-B3)** Average JPE matrices of AD patients in cohort 2. The Spearman correlation coefficients in the top right corner of each matrix quantify the consistency of connectivity patterns between cohort 1 and cohort 2 for the AD patient group. **(C1-C3)** Average JPE matrices of control subjects in cohort 1. **(D1-D3)** Average JPE matrices of control subjects in cohort 2. The Spearman correlation coefficients in the top right corner of each matrix quantify the consistency of connectivity patterns between cohort 1 and cohort 2 for the control group. Matrices are presented for the theta (4-8 Hz), alpha (8-13 Hz) and beta (13-30 Hz) frequency bands. Regions of interest (1-80) are ordered from the left to the right hemisphere (see Supplementary Table 1). All Spearman correlations had a significance level of p < .001 (FDR-corrected).

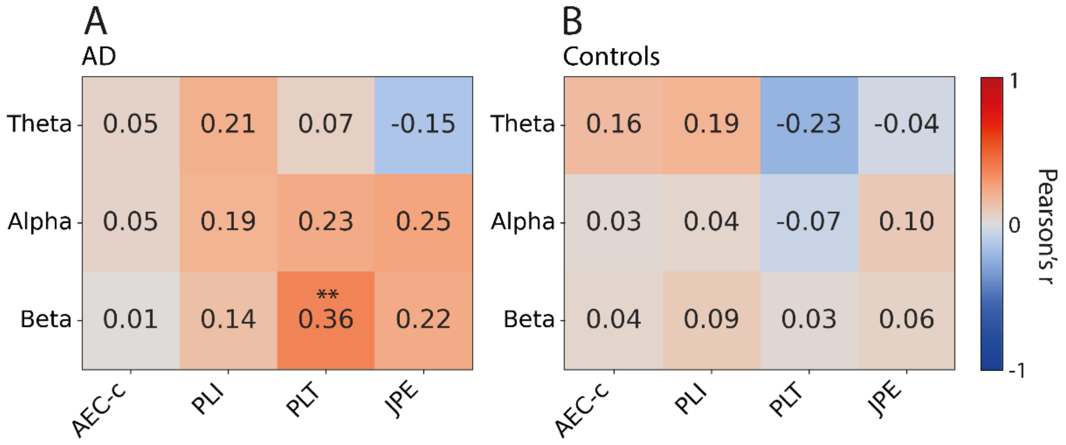

Supplementary Figure 6 Pearson correlation between whole-brain functional connectivity and age. The Pearson correlation coefficient is presented for each functional connectivity measure (AEC-c, PLI, PLT, and JPE) in each frequency band (theta (4–8 Hz), alpha (8–13 Hz), and beta (13–30 Hz)), in relation to age. Cohorts 1 and 2 were merged into a single cohort prior to computation of the correlations. **(A)** Results for AD patients. **(B)** Results for control subjects. **p < .01.

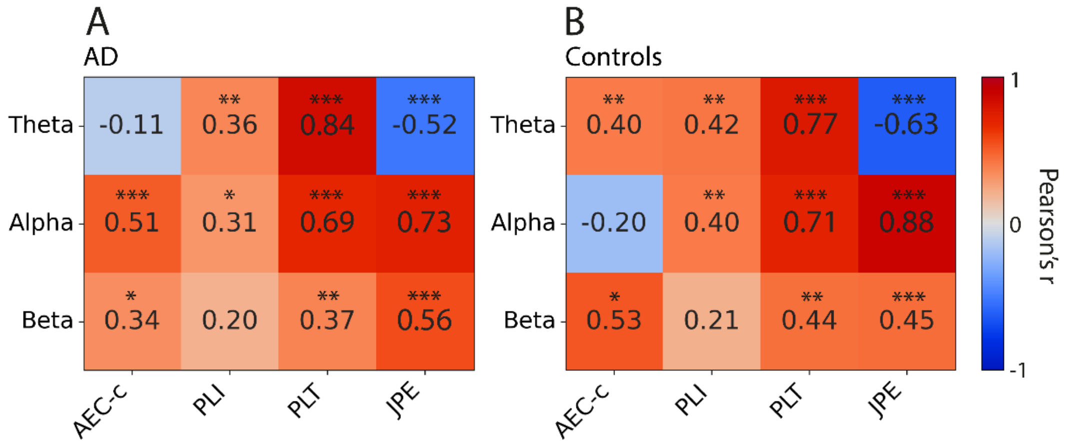

Supplementary Figure 7 Pearson correlation between whole-brain functional connectivity and whole-brain relative power in the corresponding frequency band. The Pearson correlation coefficient is presented for each functional connectivity measure (AEC-c, PLI, PLT, and JPE) in each frequency band (theta (4–8 Hz), alpha (8–13 Hz), and beta (13–30 Hz)), in relation to relative power in the corresponding frequency band. Cohorts 1 and 2 were merged into a single cohort prior to computation of the correlations. **(A)** Results for AD patients. **(B)** Results for control subjects. * p < .05; **p < .01;*** p < .001.

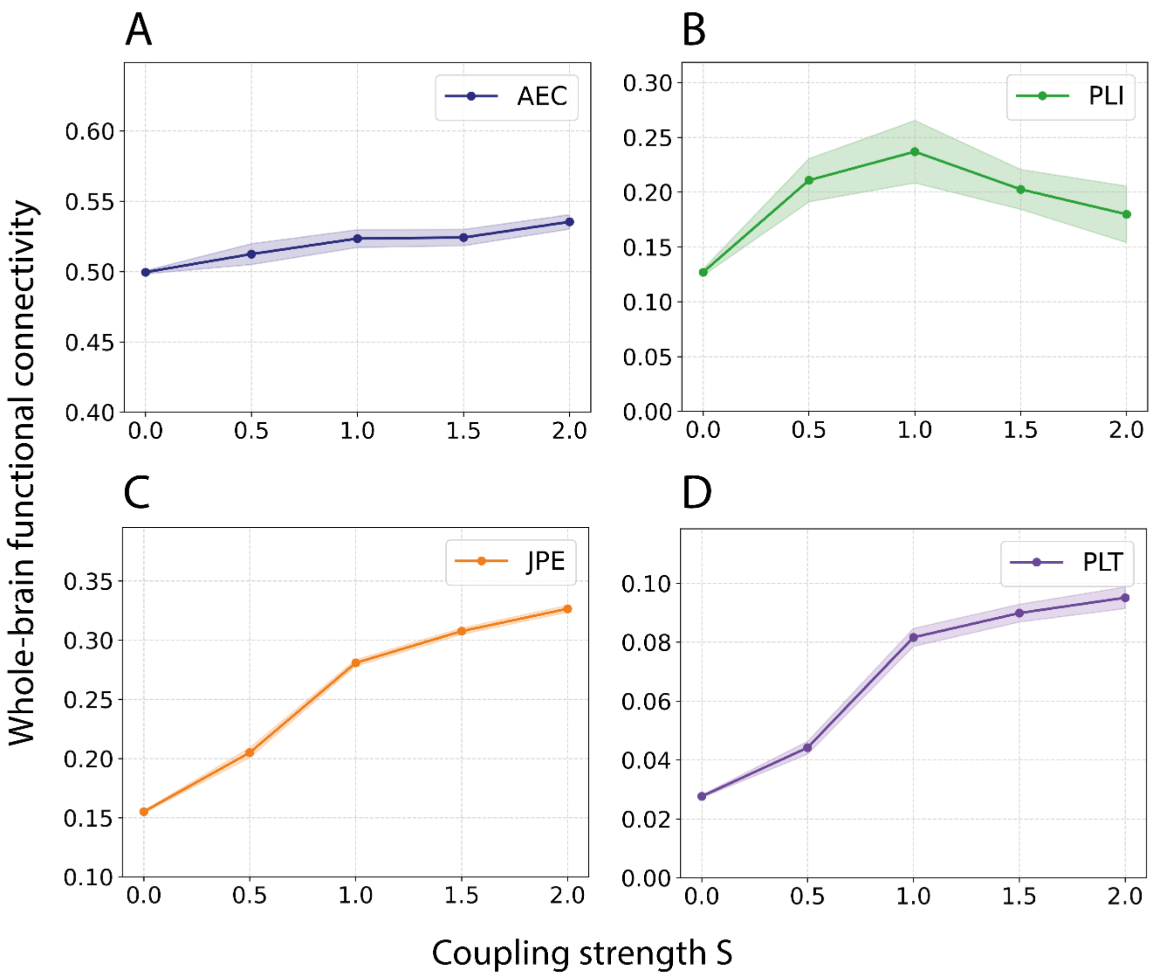

Supplementary Figure 8 Whole-brain functional connectivity as a function of coupling strength. Displayed are mean (±SD) changes in whole-brain functional connectivity estimated by **(A)** AEC, **(B)** PLI, **(C)** PLT, and **(D)** JPE, derived from simulated MEG data across varying values of global coupling strength S in the model.
